# Self-help interventions for depressive disorders and depressive symptoms: a systematic review

**DOI:** 10.1186/1744-859X-7-13

**Published:** 2008-08-19

**Authors:** Amy J Morgan, Anthony F Jorm

**Affiliations:** 1 Orygen Youth Health Research Centre, Department of Psychiatry, University of Melbourne, Parkville, Australia

## Abstract

**Background:**

Research suggests that depressive disorders exist on a continuum, with subthreshold symptoms causing considerable population burden and increasing individual risk of developing major depressive disorder. An alternative strategy to professional treatment of subthreshold depression is population promotion of effective self-help interventions that can be easily applied by an individual without professional guidance. The evidence for self-help interventions for depressive symptoms is reviewed in the present work, with the aim of identifying promising interventions that could inform future health promotion campaigns or stimulate further research.

**Methods:**

A literature search for randomised controlled trials investigating self-help interventions for depressive disorders or depressive symptoms was performed using PubMed, PsycINFO and the Cochrane Database of Systematic Reviews. Reference lists and citations of included studies were also checked. Studies were grouped into those involving participants with depressive disorders or a high level of depressive symptoms, or non-clinically depressed participants not selected for depression. A number of exclusion criteria were applied, including trials with small sample sizes and where the intervention was adjunctive to antidepressants or psychotherapy.

**Results:**

The majority of interventions searched had no relevant evidence to review. Of the 38 interventions reviewed, the ones with the best evidence of efficacy in depressive disorders were S-adenosylmethionine, St John's wort, bibliotherapy, computerised interventions, distraction, relaxation training, exercise, pleasant activities, sleep deprivation, and light therapy. A number of other interventions showed promise but had received less research attention. Research in non-clinical samples indicated immediate beneficial effects on depressed mood for distraction, exercise, humour, music, negative air ionisation, and singing; while potential for helpful longer-term effects was found for autogenic training, light therapy, omega 3 fatty acids, pets, and prayer. Many of the trials were poor quality and may not generalise to self-help without professional guidance.

**Conclusion:**

A number of self-help interventions have promising evidence for reducing subthreshold depressive symptoms. Other forms of evidence such as expert consensus may be more appropriate for interventions that are not feasible to evaluate in randomised controlled trials. There needs to be evaluation of whether promotion to the public of effective self-help strategies for subthreshold depressive symptoms could delay or prevent onset of depressive illness, reduce functional impairment, and prevent progression to other undesirable outcomes such as harmful use of substances.

## Background

Data from recent epidemiological studies suggest that depressive disorders exist on a continuum, rather than in separate categories [1,2]. As a consequence, research has begun to accumulate on the clinical relevance and public health significance of depressive symptoms not meeting diagnostic criteria, variously labelled subthreshold, subclinical, subsyndromal, mild, or minor depression. Here, we use the term subthreshold depression. Subthreshold depression is prevalent [3], increases the risk of developing major depressive disorder [4], and has considerable economic costs [5]. At the individual level, disability from subthreshold depression is lower than for depressive disorders; however, the burden of disability for the population as a whole is substantial for subthreshold depression because of its greater prevalence [6]. Given that unipolar depressive disorders were the leading cause of disability burden globally in 2001 [7], depressive symptoms falling short of a disorder are of major public health significance.

Several trials have investigated treatments for milder depressive states, with some success [3,8]. However these treatments, which include antidepressant medication and brief psychotherapy, involve the participation of health professionals. An approach that does not further burden clinical resources is preferable, as there is already a large group of people with major depression who do not receive treatment [9], and treating these people deserves priority over those with subthreshold symptoms. An alternative approach is self-help that can be applied by the individuals affected without the need for professional guidance.

Self-help approaches for depression are commonly used, particularly for milder forms of depression [10,11], and are perceived as helpful by the public [12]. However, some self-help methods in common use are probably self-defeating (for example, substance use). If effective informal self-help methods could be identified, they could be used as a cost-effective way of reducing subthreshold depressive symptoms. Health promotion campaigns on other major sources of disease burden, such as heart disease and cancer, routinely include information on actions that can be taken to reduce risk. Jorm and Griffiths [13] called for this approach to be extended to self-help interventions for depression, with the aim of reducing subthreshold depressive symptoms and the risk of progressing to a depressive disorder. If applied successfully, such an approach would have the potential to reduce the distribution of symptoms across the whole population. However, due to the risk of suicide and detriment to functioning if symptoms deteriorate or do not improve, such an approach would also need clear guidelines on when to seek professional help rather than relying on self-help strategies.

If a health promotion approach were to be applied, the first step is to identify a small number of self-help actions that are likely to be effective and that can be applied easily by many people at low cost. A number of reviews have examined the evidence for self-help or complementary therapies for depression [14-19]. These have found reasonable evidence for St John's wort, S-adenosylmethionine, exercise, bibliotherapy, and light therapy. Although these reviews are informative, we decided to undertake our own systematic review of the evidence because prior reviews were either outdated (in a rapidly growing research area), only reviewed treatments for depressive disorders and not subthreshold symptoms, or they focused solely on complementary and alternative therapies rather than other self-help strategies.

## Methods

### Selection of treatments to review

Treatments were identified from previous systematic reviews of complementary and self-help treatments for depression [14,19]. Not all of these treatments were included for review here as some required the assistance of another person (for example, LeShan distance healing) or a visit to a practitioner (for example, acupuncture).

### Search methodology

PubMed, PsycINFO and the Cochrane Database of Systematic Reviews were searched using the following terms: name-of-treatment (and synonyms) AND (depressi* OR dysthym* OR affective OR mood), limited to English and humans (see Additional file 1 for search details). Most searches were carried out of literature up to March 2007, however a few treatments found in the course of the review were searched up to September 2007. Reference lists and citations of included studies were also checked. Treatments with no relevant studies to review are listed in Table 1. Studies were reviewed by one author and the accuracy of each review was checked by a second.

**Table 1:** Self-help methods with no relevant trials to review

**Category**	**Treatment**
Medicines/herbs/dietary supplements	5-hydroxytryptophan, American ginseng (*Panax quinquefolius*), ashwaganda (*Withania somnifera*), astragalus (*Astragalus membranaceous*), Bach flower remedies, basil (*Ocimum spp*.), black cohosh (*Actaea racemosa *and *Cimicifuga racemosa*), brahmi (*Bacopa monniera*), California poppy (*Eschscholtzia californica*), catnip (*Nepeta cataria*), cat's claw (*Uncaria tomentose*), chamomile (*Anthemis nobilis*), chaste tree berry (*Vitex agnus castus*), chocolate, choline, clove (*Eugenia caryophyllata*), coenzyme Q_10_, combined preparations (Empowerplus (Truehope Nutritional Support Ltd.); euphytose; Mindsoothe Jr. (Native Remedies); Sedariston; Worry Free), cowslip (*Primula veris*), damiana (*Turnera diffusa*), dandelion (*Taraxacum officinale*), flax seeds (linseed) (*Linum usitatissimum*), Gamma-aminobutyric acid (GABA), ginger (*Zingiber officinale*), gotu kola (*Centella asiatica*), glutamine, hawthorn (*Crataegus laevigata*), hops (*Humulus lupulus*), hyssop (*Hyssopus officinalis*), inositol, Kava (*Piper methysticum*), lemon balm (*Melissa officinalis*), lemongrass leaves (*Cymbopogon citrates*), liquorice (*Glycyrrhiza glabra*), magnesium, milk thistle (*Silybum marianum*), mistletoe (*Viscum album*), motherwort (*Leonurus cardiaca*), natural progesterone, nettles (*Urtica dioica*), oats (*Avena sativa*), painkillers/over the counter medicines, para-aminobenzoic acid (PABA), passionflower (*Passiflora incarnata*), peppermint (*Mentha piperita*), phenylalanine, potassium, purslane (*Portulaca oleracea*), rehmannia (*Rehmannia glutinosa*), Rhodiola rosea, rosemary (*Rosmarinus officinalis*), sage (*Salvia officinalis*), schizandra (*Schizandra chinensis*), Siberian ginseng (*Eleutherococcus senticosus*), skullcap (*Scutellaria lateriflora*), spirulina (*Arthrospira platensis*), St Ignatius bean (*Ignatia amara*), taurine, tension tamer, thyme (*Thymus vulgaris*), tissue salts, tyrosine, valerian (*Valeriana officinalis*), vervain (*Verbena officinalis*), vitamin B_2_, vitamin B_3_, vitamin B_5_, vitamin B_7_, vitamin E, vitamin K, wild yam (*Dioscorea villosa*), wood betony (*Stachys officinalis*; *Betonica officinalis*), yeast, zinc, zizyphus (*Zizyphus spinosa*)
Dietary methods	Avoiding barley, rye, sugar, wheat, or dairy foods, ketogenic diet
Substances	Drinking or reducing alcohol consumption, using cannabis or quitting cannabis, smoking a cigarette or quitting smoking
Lifestyle changes	Adequate sleep, holiday or vacation, pilates, recreational dance, shopping
Physical and sensory methods	Crystal healing or charm stone, fragrance, reflexology

### Inclusion/exclusion criteria

Studies were included for review if they evaluated the treatment's effects on depression symptoms or depressed mood, using a reliable and valid scale for depression or depressed mood. In contrast with the previous reviews which only included studies with individuals selected to have a depressive disorder or a high level of depressive symptoms, in this review we also included studies with participants not selected for depression, as they may have had subthreshold or mild depression symptoms. Studies were grouped as involving depressive disorders (participants with a depressive disorder or a high level of depressive symptoms) or non-clinically depressed (participants not selected for depression). The scope of the review was limited to randomised controlled trials with sufficiently large samples that had the power to detect a standardised mean difference (d) of 1. Studies with independent groups were rejected if they had less than 17 participants per group (this gives 80% power to detect an effect size of d = 1 in independent groups with alpha set at 0.05) and crossover studies were rejected if there were less than 10 participants (assuming a correlation of 0.5 between ratings, this gives 80% power to detect an effect size of d = 1 with alpha set at 0.05). Trials without an appropriate control intervention or with uninterpretable findings were also excluded. No age restrictions were applied, but studies with children/adolescents, adults, or older adults were reviewed separately where appropriate. Preference was given to reviewing recent meta-analyses or systematic reviews where they were available. As we were interested in interventions that could be applied by most individuals with depression, and without recourse to a professional, studies were excluded from the review if:

• the self-help treatment was in addition to an antidepressant or psychotherapy (adjunctive or augmentation studies);

• participants had a comorbid medical or mental illness with depression secondary;

• participants were primarily bipolar patients;

• they investigated premenstrual syndrome/premenstrual dysphoric disorder, postpartum depression, or hormone-related depression (for example, in menopausal women);

• the depression symptoms were caused by a clear underlying nutritional deficiency (for example, magnesium) or underlying medical condition (for example, coeliacs disease or mitochondrial disorder).

## Results

There were 38 interventions with relevant evidence to review. For convenience, interventions have been grouped under the categories of herbal remedies or dietary supplements, substances, dietary methods, psychological methods, lifestyle changes, and physical and sensory methods. For some interventions, no evidence regarding effects on depression was available (see Table 1).

## Herbal remedies or dietary supplements

### Borage (Borago officinalis or Echium amoenum)

#### Description and rationale

Borage is a herb originating in Syria. The flowers of the plant can be used in herbal teas. Although the plant is used in traditional Iranian medicine for mood enhancement, its antidepressant mechanism is unclear.

#### Review of efficacy

##### Depressive disorders

There has been one small randomised controlled trial (RCT) [20]. A total of 35 adults with mild to moderate major depressive disorder received either placebo or 375 mg of aqueous extract of borage flowers daily for 6 weeks. By week 4 there was a small significant difference in levels of depression symptoms between the two groups, with lower levels in the borage group. Results at week 6 were similar but no longer statistically significant.

#### Conclusion

There is preliminary evidence that borage flower extract may be helpful for depression. Longer trials with larger samples are needed to confirm these results. There is no evidence on the effects of borage in non-clinically depressed people.

### Carnitine/acetyl-L-carnitine

#### Description and rationale

Carnitine is a nutrient involved in energy metabolism. It is produced in the body and is available in food such as meat and dairy products. Acetyl-L-carnitine is an ester of carnitine that readily enters the brain. Carnitine supplements are available from pharmacies and health food shops. The antidepressant mechanism is unknown. Possible mechanisms include an inhibitory effect on the hypothalamic-pituitary-adrenal axis activity resulting in a reduction of cortisol levels [21] or effects on membrane phospholipid metabolism and membrane physical/chemical properties [22].

#### Review of efficacy

##### Depressive disorders

Three RCTs have evaluated acetyl-L-carnitine supplementation in individuals with dysthymia [23-25]. Of these trials, 2 were in 46 or 52 older adults (aged 60 to 80 years) and compared 3 g daily doses of acetyl-L-carnitine with placebo over 60 days. Those taking acetyl-L-carnitine showed significantly improved depression symptoms compared with those taking placebo. The other trial compared 1 g daily dosage of acetyl-L-carnitine with 50 mg amisulpride in 193 participants with dysthymia and found both groups had improved depression symptoms over 3 months and there was no significant difference in improvement between groups [25].

##### Non-clinically depressed

A double-blind RCT evaluated the effect over days of carnitine on depressed mood [26]. A total of 400 adult females received either a placebo, 2 g carnitine, 1.6 g lecithin, or both lecithin and carnitine for 3 days. Carnitine supplementation had no effect on depressed mood.

#### Conclusion

Preliminary evidence suggests acetyl-L-carnitine may be helpful for dysthymia, particularly in older adults. From the limited evidence available carnitine does not appear to be effective in non-clinically depressed adults.

### Chromium

#### Description and rationale

Chromium is an essential trace mineral involved in carbohydrate, fat and protein metabolism. Chromium is available in food or as a supplement from health food shops, usually in the form chromium picolinate. The antidepressant mechanism is unknown but could involve increased insulin sensitivity resulting in enhanced central noradrenergic and serotonergic activity [27].

#### Review of efficacy

##### Depressive disorders

A trial of 113 adults with atypical depression who took either 400 to 600 μg chromium picolinate or placebo for 8 weeks found no significant difference in the reduction of depression symptoms or rates of response [28]. However, a subgroup analysis found that adults who had high carbohydrate craving showed a greater response to chromium than placebo.

#### Conclusion

Limited evidence suggests chromium supplementation is not helpful for depressive disorders, although there is tentative evidence that it may be helpful for a subgroup of atypically depressed adults with high levels of carbohydrate craving. There is no evidence on the effects of chromium in non-clinically depressed people.

### Ginkgo biloba

#### Description and rationale

Extracts from the leaves of the Ginkgo biloba (maidenhair) tree are available in tablet form from health food shops. Its antidepressant mechanism is proposed to be a reduction in the production of stress hormones [29]. Ginkgo may also be effective for the treatment of impaired cerebral circulation in the elderly, one symptom of which is depressed mood [30].

#### Review of efficacy

##### Non-clinically depressed

Two RCTs in 104 healthy young adults and 93 older adults of 120 mg ginkgo daily for 12 weeks showed no effect on depressed mood [31].

#### Conclusion

From the limited evidence available, ginkgo does not appear effective for depressed mood in non-clinically depressed adults. There is no evidence on the effects of ginkgo on depressive disorders.

### Korean ginseng (Panax ginseng)

#### Description and rationale

Korean ginseng is a herb native to Korea and China. Extracts from the root of the plant are available as supplements from health food shops. The major active constituents are thought to be ginsenosides which may increase resistance to stress through their action on the hypothalamic-pituitary-adrenal axis [32].

#### Review of efficacy

##### Non-clinically depressed

One RCT has examined ginseng's effects on mood over months in healthy adults. In all, 83 participants took either 200 mg ginseng, 400 mg ginseng or placebo daily for 60 days [33]. Ginseng supplementation had no effect on depressed mood.

#### Conclusion

From the limited evidence available, ginseng does not appear to be effective for depressed mood in non-clinically depressed individuals. There is no evidence on the effects of ginseng on depressive disorders.

### Lavender (Lavandula angustifolia)

#### Description and rationale

Lavender is a traditional herbal remedy that is thought to 'strengthen the nervous system' [34] and may aid sleep and relaxation. Extracts are obtained from the flowering tops.

#### Review of efficacy

##### Depressive disorders

One small double-blind RCT has compared lavender with an antidepressant in adults with depressive disorders [34]. A total of 45 adults with major depression participated in a 4-week trial where they received 60 drops of a lavender tincture plus placebo tablet, 100 mg imipramine plus placebo drops, or lavender plus imipramine. Although depression symptoms improved significantly in all groups, the lavender group improved significantly less than the imipramine group, and there was no placebo control group to rule out placebo effects.

#### Conclusion

There is insufficient evidence to determine whether lavender may be helpful for depressive disorders. There is no evidence on the effects of lavender in non-clinically depressed people.

### Lecithin

#### Description and rationale

Lecithin is a mixture of phospholipids and is a major component of cell membranes. Lecithin is found in foods such as eggs and soy beans, but is also available as a supplement from health food shops. Choline, a component of lecithin, is a precursor to acetylcholine, which is needed for normal brain functioning.

#### Review of efficacy

##### Non-clinically depressed

One double-blind RCT has examined the effect over days of lecithin on depressed mood [26]. A total of 400 adult females received either a placebo, 1.6 g lecithin (phosphatidylcholine), 2 g carnitine, or both lecithin and carnitine for 3 days. Lecithin supplementation had no effect on depressed mood.

#### Conclusion

From the limited evidence available lecithin does not appear to be effective for depressed mood in non-clinically depressed individuals. There is no evidence on the effects of lecithin on depressive disorders.

### Melatonin

#### Description and rationale

Melatonin is a hormone involved in the regulation of sleep/wake cycles. Over the counter supplements are available in some countries. The mechanism is unclear, but research suggests melatonin production is disturbed in depressed people, and that a dysfunction in the timing of melatonin production is a possible cause of seasonal affective disorder [35].

#### Review of efficacy

##### Non-clinically depressed

An RCT of 53 adults with subsyndromal SAD and/or weather-associated syndrome who took 2 mg slow-release melatonin in the evening for 3 weeks found no significant difference in atypical depression symptoms between melatonin and placebo [36].

#### Conclusion

Limited evidence suggests that melatonin has no effect on depressive symptoms in non-clinically depressed individuals. There is no evidence on the effects of melatonin on depressive disorders.

### Omega 3 fatty acids (fish oils)

#### Description and rationale

Omega 3 fatty acids are long-chain polyunsaturated fatty acids. The two most important for depression are eicosapentanoic acid (EPA) and docosahexanoic acid (DHA), which are found in fish or are made in the body from alpha-linolenic acid (another omega 3 fatty acid, found in flaxseed, walnuts and canola oil). Omega 3 supplements (containing EPA and DHA) are available from health food shops and pharmacies. Several lines of evidence suggest a link between omega 3 fatty acids and depression. An increase in rates of depression in Western countries has paralleled a change in diet to one favouring omega 6 over omega 3 fatty acids; across countries there is a strong negative association between fish consumption and depression; and lower concentrations of omega 3 have been found in the blood of depressed people. Possible mechanisms include omega 3's effects on the fluidity of cell membranes, which leads to changes in signalling within and between brain cells; and omega 3's anti-inflammatory effects, as depression may be caused by an overactive inflammation response.

#### Review of efficacy

##### Depressive disorders

Although there have been several reviews of omega 3 fatty acids for depression [37,38], only one study has evaluated omega 3 as a single treatment for depression in sufficient participants [39]. A double-blind RCT of 35 depressed adults with low fish intake who took 2 g DHA or placebo daily for 6 weeks found that omega 3 supplementation was no better than placebo in reducing depression symptoms.

##### Non-clinically depressed

A single RCT of 49 healthy adults examined the effect on depressed mood of supplementation of 4 g fish oil (containing 1,600 mg EPA and 800 mg DHA), or placebo for 35 days [40]. Depressed mood reduced significantly in the omega 3 group but not in the placebo group.

#### Conclusion

The only trial to qualify for inclusion in the review found that omega 3 fatty acids were not helpful for depressive disorders. Preliminary evidence suggests omega 3 fatty acids for depressed mood in non-clinically depressed individuals may be beneficial, but requires replication in further trials.

### S-Adenosylmethionine

#### Description and rationale

S-Adenosylmethionine (SAMe) is a compound that is manufactured in the body, is a major methyl donor in the brain and is involved in many biochemical reactions. Supplements are available in a number of countries from pharmacies and health food shops. The antidepressant mechanism of SAMe is unknown, but may involve its effects on the fluidity of neuronal membranes or its involvement in serotonin, dopamine and norepinephrine synthesis.

#### Review of efficacy

##### Depressive disorders

Both a recent systematic review [41] and a meta-analysis [42] have found SAMe helpful for depressive disorders. The systematic review was restricted to trials that passed a quality assessment. Those included were five uncontrolled trials and two RCTs. Despite differences in doses, route of administration (oral, intramuscular, intravenous) and comparison or control treatments, SAMe had a consistent positive effect over weeks or months. An additional RCT was included after the review was completed, which found that the efficacy of 1,600 mg/day oral SAMe or 400 mg/day intramuscular SAMe was not significantly different from 150 mg/day of imipramine. The meta-analysis included 28 trials and found greater improvement with SAMe than with placebo (global effect size ranging from 17% to 38% depending on definition of response), and no difference in outcomes between treatment with SAMe and standard tricyclic antidepressants.

#### Conclusion

There is consistent evidence that SAMe may be helpful for depressive disorders in adults. Further large, longer-term RCTs are needed to clarify questions regarding optimum dosage, safety and comparison with newer antidepressants. An RCT in children and adolescents is warranted. There is no evidence on the effects of SAMe in non-clinically depressed people.

### Saffron (*Crocus sativus *L.)

#### Description and rationale

Saffron is the world's most expensive spice, made from the stigma of the flower of the *Crocus sativus*. Both the stigma and the petal (which is much cheaper) have been used for the treatment of depression. Saffron is used for depression in Persian traditional medicine. Its mechanism is unclear, but it has been proposed that two components of saffron, crocin and safranal, inhibit reuptake of dopamine, norepinephrine and serotonin [43].

#### Review of efficacy

##### Depressive disorders

Four double-blind RCTs have examined the effect of saffron (stigma) or Crocus sativus petals on depressed adults. Two trials each with 40 adults compared saffron stigma (30 mg daily), with fluoxetine (20 mg) [44] or with placebo [45] for 6 weeks. Saffron significantly reduced depression symptoms more than placebo, and there was no significant difference in efficacy between saffron and fluoxetine. Similarly, 30 mg extracts from the petals of Crocus sativus have also shown efficacy similar to 20 mg fluoxetine [46] and greater efficacy than placebo [47] in trials of 40 adults.

#### Conclusion

Evidence for the efficacy of saffron in adults with depressive disorders is promising. The results need to be replicated by other research groups in larger trials with longer durations. There is no evidence on the effects of saffron in non-clinically depressed people.

### Selenium

#### Description and rationale

Selenium is an essential trace element although it can be toxic in high doses. Selenium is found in high protein foods, or is available as a supplement from health food shops. Although it is preferentially retained in the brain during times of deficiency, no mechanism has been proposed for how it might affect mood.

#### Review of efficacy

##### Non-clinically depressed

Two trials have examined selenium intake and depressed mood in non-depressed adults. A double-blind crossover trial found daily supplementation of 100 μg selenium in 50 adults significantly improved depressed mood over 5 weeks (compared to placebo) [48,49] and a RCT found no effect of a range of dosages of selenium supplementation in 448 older adults over 6 months [50].

#### Conclusion

Evidence for selenium's effect on depressed mood in non-clinically depressed adults is inconsistent. Although one trial found an effect, the larger and better designed study did not. There is no evidence on the effects of selenium on depressive disorders.

### St John's wort (*Hypericum perforatum*)

#### Description and rationale

St John's wort is a traditional herbal remedy for depression. It is widely available as a supplement from health food shops, pharmacies and supermarkets. The most important active compounds are believed to be hypericin and hyperforin, but other compounds may also play a role. How it works is still not entirely clear, however it may inhibit the uptake of serotonin, norepinephrine, and dopamine [51].

#### Review of efficacy

##### Depressive disorders

Several systematic reviews and meta-analyses examining St John's wort for depression have been published in recent times. A systematic review of these reviews [51] concluded that although review methodologies have varied, St John's wort has consistently been found to be beneficial for mild to moderate depression compared to placebo, although the degree of benefit has varied between reviews. Comparisons against antidepressants have usually found no difference in benefit. The most recent Cochrane review of St John's wort for depression, published after the above mentioned review was completed, paints a more complex picture [52]. The review was restricted to double blind RCTs of at least 4 weeks duration in adults with depressive disorders. A total of 37 trials involving 4,925 participants met inclusion criteria, and the majority were judged reasonable to good quality. Pooled results from 24 trials found that St John's wort was overall superior to placebo (response rate ratio 1.55, 95% confidence interval (CI) 1.42 to 1.70), and pooled results from 13 trials found no difference between St John's wort and older or newer antidepressants (response rate ratio 1.01, 95% CI 0.93 to 1.10). However, results from the studies comparing St John's wort to placebo were heterogeneous, with metaregression analyses leading to the conclusion that St John's wort showed greater benefits for individuals with mild depression. A variety of preparations of St John's wort were used and daily doses ranged from 240 mg to 1,800 mg. St John's wort caused fewer negative side effects than older antidepressants, and may have caused slightly fewer negative side effects than newer antidepressants. Use of St John's wort is not without risk however, as it has the potential to make other medications (such as immune suppressants, oral contraceptives and anticoagulants) less effective by increasing their rate of metabolism, and can also interact with selective serotonin reuptake inhibitors to cause a toxic reaction [51].

#### Conclusion

St John's wort for depressive disorders has been well researched and there is evidence that it is helpful for mild depression. Consumers should be aware of risks involved when taken with other medications, and the possibility of variable quality of extracts in different brands and batches. There is no evidence on the effects of St John's wort in non-clinically depressed people.

### Vitamins

#### Description and rationale

Vitamins may play a role in depression or depressed mood because the brain depends on a constant supply to function effectively, and subclinical deficiencies are relatively common [53]. Thiamine is required for the synthesis of acetylcholine. Vitamin B_6 _is a cofactor for the decarboxylases involved in the synthesis of neurotransmitters GABA, dopamine, norepinephrine, serotonin and histamine [54]. Folic acid and vitamin B_12 _are coenzymes for catechol-O-methyl transferase important in the breakdown of catecholamines. Vitamin C is necessary for the synthesis of dopamine and norepinephrine [55]. As vitamin D levels decrease during winter due to reduced sunlight exposure, low levels of vitamin D may play a role in winter depression (seasonal affective disorder).

### Review of efficacy

#### Vitamin B_1 _(thiamine)

##### Non-clinically depressed

A double-blind RCT in 117 healthy young adult females of 50 mg thiamine or placebo daily for 2 months found that supplementation had no effect on depressed mood [56].

#### Vitamin B_6_

##### Depressive disorders

Although a systematic review has examined vitamin B_6 _for depression [57], all trials evaluated vitamin B_6 _in combination with another treatment or used it only with hormone-related depression.

##### Non-clinically depressed

A single double-blind RCT has been carried out in 211 young, middle-aged and older female adults of 75 mg vitamin B_6_, 750 μg folate, 15 μg vitamin B_12 _or placebo for 5 weeks. Vitamin B_6 _supplementation had no effect on depression symptoms or depressed mood [53].

#### Vitamin B_12_

##### Non-clinically depressed

Two double-blind RCTs have tested the effect of supplementation of vitamin B_12 _on depression symptoms in healthy adults. A weekly injection of 1 mg B_12 _for 4 weeks in 134 elderly adults who showed signs of vitamin deficiency did not reduce depression symptoms significantly more than placebo [58]. Similarly, B_12 _had no effect on depression symptoms or depressed mood when taken daily in a dose of 15 μg for 5 weeks in a double-blind RCT of 211 young, middle-aged and older female adults [53].

#### Folate

##### Depressive disorders

Although a systematic review examined folate for depression [59] no RCTs were included that examined folate on its own as a treatment for people who were depressed without other medical conditions.

##### Non-clinically depressed

A double-blind RCT in 211 young, middle-aged and older female adults of 750 μg folate, 15 μg vitamin B_12_, 75 mg vitamin B_6 _or placebo for 5 weeks found folate supplementation had no effect on depression symptoms or depressed mood [53].

#### Vitamin C

##### Non-clinically depressed

A double-blind RCT in 81 healthy young adults who took 3,000 mg sustained-release vitamin C or placebo for 14 days found depression symptoms significantly decreased in the vitamin C but not the placebo group [60]. However, the decrease was very small.

#### Vitamin D

##### Non-clinically depressed

Three RCTs have examined vitamin D supplementation in healthy adults. In all, 250 middle-aged and older adult females took 377 mg calcium plus 400 IU vitamin D daily, or 377 mg calcium on its own for a year [61]. Depressed mood was assessed four times over the year, with vitamin D showing no effect. A 5-day trial in 44 adults of either vitamin D (400 IU or 800 IU) plus vitamin A (9,000 IU or 8,000 IU) versus 10,000 IU vitamin A only, found that vitamin D improved positive mood, but did not change negative mood [62]. Finally, a large 6-month trial of 2,117 women aged over 70 years compared supplementation with vitamin D (800 IU) plus calcium (1,000 mg) with no supplementation. No significant difference was found in depressed mood between the two groups [63].

#### Multivitamins

##### Non-clinically depressed

Seven double-blind RCTs have examined the effects of multivitamin supplementation on depressed mood or symptoms in healthy non-depressed adults. A total of 120 young adults took a placebo or a multivitamin that contained 10 times the US recommended daily amount except for vitamin A (3,334 IU vitamin A, 14 mg B_1_, 16 mg B_2_, 180 mg B_3_, 22 mg B_6_, 2 mg B_7_, 0.03 mg B_12_, 600 mg vitamin C, 100 mg vitamin E and 4 mg folate), for 12 months [55]. Supplementation had no effect on depressed mood after 3 or 12 months. A similar trial in 126 older adults of supplementation of the same multivitamin combination and dosage for 24 weeks also had no effect on depressed mood [54]. A trial in 95 adults of Pharmaton capsules (a supplement containing vitamins, minerals, trace elements and ginseng) for 8 weeks showed no effect on depressed mood [64]. A larger follow-up trial in 313 adults of the same supplement for 8 weeks also showed no effect on depressed mood. However, a subgroup analysis found that participants who were dieting had a greater improvement in depressed mood if they were taking the supplement than if they were taking the placebo [65]. A trial in 77 adult males of Berocca Performance supplementation (containing vitamins B_1_, B_2_, B_3_, B_5_, B_6_, B_7_, B_12_, folate, C, and calcium, magnesium, zinc) for 28 days found that Berocca was no better than placebo at reducing depression symptoms [66]. Finally, a trial of antioxidant supplementation (consisting of 12 mg/day β-carotene, 400 mg/day α-tocopherol, and 500 mg/day vitamin C) in 185 older adults for 12 months also showed no effect on depressed mood [67].

#### Conclusion

The limited evidence suggests that thiamine, vitamin B_6_, vitamin B_12 _and folate supplementation are not helpful for depressed mood or symptoms in non-clinically depressed individuals. The evidence for vitamin D in non-clinically depressed individuals is inconsistent, but the larger, longer trials suggest it is not helpful. The evidence is more conclusive that multivitamins are not helpful for depressed mood in non-clinically depressed people. However, limited evidence suggests that vitamin C may be helpful in non-clinically depressed individuals, but these results require replication.

## Substances

### Caffeine

#### Description and rationale

Caffeine is a central nervous system stimulant that blocks adenosine receptors, which causes an increase in the levels of several neurotransmitters including dopamine and serotonin [68]. Caffeine consumption is associated with depression symptoms. This may be because depressed individuals self-treat with caffeine [69]. However, large doses can produce anxiety symptoms.

#### Review of efficacy

##### Non-clinically depressed

A review of studies, including several RCTs that evaluated caffeine consumption in healthy adults generally concluded that caffeine temporarily improves feelings of wellbeing, energy and mood [69]. However, caffeine use is widespread, study participants are typically not allowed caffeine before the experiment, and withdrawal from caffeine often involves depressed mood [70]. Therefore some argue that the mood benefits are due to a reversal of withdrawal symptoms [71]. Others disagree with this interpretation and argue that positive effects of caffeine on mood have been found when participants were not in caffeine withdrawal [69].

#### Conclusion

Although consumption of caffeine appears to improve depressed mood in non-clinically depressed individuals, it is still unclear whether this is caused by a reversal of withdrawal symptoms or is a true effect. There is no evidence on the effects of caffeine on depressive disorders.

## Dietary methods

### Carbohydrate-rich, protein-poor meals

#### Description and rationale

It has been suggested that a meal rich in carbohydrates but low in protein lifts mood, and that some depressed people (particularly those with seasonal affective disorder) could increase their carbohydrate intake in order to relieve depressive symptoms. The proposed mechanism is that a meal almost exclusively carbohydrate increases the level of tryptophan transported into the brain, where it is then converted into serotonin. However, most high-carbohydrate meals contain sufficient protein to block this mechanism [72].

#### Review of efficacy

##### Depressive disorders

A crossover trial has compared the effects on depressed mood over hours of a carbohydrate-rich, protein-poor meal with a protein-rich, carbohydrate-poor meal in 16 adults with seasonal affective disorder and 16 non-depressed adults [73]. Participants ate each meal on separate days, with the order of meals randomised. Results are difficult to interpret due to order effects. Both types of meals reduced depressed mood when eaten first, but when they were eaten second, the carbohydrate-rich meal decreased depressed mood while the protein-rich meal increased it.

##### Non-clinically depressed

Three RCTs have examined the effect over minutes or hours of a carbohydrate-rich, protein-poor meal on depressed mood. One trial found depressed mood decreased across all participants after a carbohydrate-rich meal [74], one trial found depressed mood did not increase under stressful conditions in high-stress prone individuals after the consumption of a carbohydrate-rich meal compared with a protein-rich meal [75], and one trial found no significant difference in depressed mood between a carbohydrate-rich and a protein-rich meal [76]. Studies varied in the type of participants (young adults, older adults, obese adults), time of day when meal was eaten (lunch time, early or mid afternoon), type of meal (such as cake or liquid) and the carbohydrate, fat and protein proportions of meals classified carbohydrate-rich and protein-rich.

#### Conclusion

Studies have varied methodologies and inconsistent results, making it difficult to determine whether a carbohydrate-rich, protein-poor meal improves depressed mood in people with or without a depressive disorder. Given that the proposed mechanism is unlikely to account for any effect, another mechanism, such as palatability, may be behind any effects found. In any case, the strategy would only be helpful for short-term use, as a diet low in protein would reduce the dietary source of tryptophan.

## Psychological methods

### Autogenic training

#### Description and rationale

Autogenic training is the regular practice of simple mental exercises in body awareness which aim to promote relaxation and stress relief. The exercises involve passive concentration on breathing, heartbeat and warmth and heaviness of body parts. Books and websites that teach autogenic training are available. Autogenic training may be helpful for depression because it aims to teach self-regulation of autonomic nervous system processes.

#### Review of efficacy

##### Depressive disorders

A quasi-RCT compared autogenic training with psychotherapy and delayed treatment in 55 adults with depressive disorders [77]. Participants undertook weekly group autogenic training sessions plus twice-daily practice or weekly individual psychotherapy for 10 weeks. Depression symptoms in the autogenic training group improved significantly more than in the delayed-treatment control group, but significantly less than in the psychotherapy group.

##### Non-clinically depressed

One RCT allocated 134 adults with minor psychological problems to 3 months of individual autogenic training with a therapist plus twice-daily practice or a delayed-treatment group [78]. The autogenic training group had significantly improved depressed mood after 3 months, whereas the control group showed no change.

#### Conclusion

Preliminary evidence for autogenic training appears promising. However, these results have been achieved under the guidance of a therapist, and the helpfulness of self-taught autogenic therapy has not been evaluated.

### Bibliotherapy

#### Description and rationale

Bibliotherapy is a form of self help that uses structured written materials, such as books. The books present a treatment program, usually based on cognitive behaviour therapy, which encourage the reader to make changes leading to improved self-management. Two self-help books for depression that have been evaluated in trials and are available in bookstores are *Feeling good *[79] and *Control your depression *[80]. Other similar books that have not been evaluated specifically, but may be helpful [81], are *Mind over mood *[82], *Overcoming depression *[83], and *Overcoming depression: a five areas approach *[84].

#### Review of efficacy

##### Depressive disorders

Several meta-analyses have evaluated the helpfulness of bibliotherapy for depression. A recent meta-analysis pooled results from 17 trials (16 RCTs) which compared bibliotherapy with a delayed treatment control group [85]. Trial participants varied in age from adolescents to the elderly and usually had mild to moderate depression without other physical or mental health problems. Trials lasted for 7 weeks on average. The meta-analysis found bibliotherapy more effective than controls (d = 0.77, 95% CI 0.61 to 0.94). Another meta-analysis of six RCTs that used the book *Feeling good *also found a large difference in depression over 4 weeks in favour of bibliotherapy over delayed treatment (standardised mean difference = -1.36, 95% CI -1.76 to -0.96) [81]. However, the trials were small and of limited quality. An earlier meta-analysis of four RCTs, which compared bibliotherapy with individual therapy, found no significant difference in depression [86]. The trials used different kinds of bibliotherapy, had small samples, and lasted between 6 and 11 weeks.

#### Conclusion

Evidence suggests that bibliotherapy is helpful for depressive disorders. However a number of caveats should be noted. The trials have not evaluated the use of bibliotherapy in the absence of any professional involvement. Also, not everyone may benefit from bibliotherapy; there are those who may lack the concentration or motivation required, have insufficient reading skills, or not be suited for personality reasons. There is no evidence on the effects of bibliotherapy in non-clinically depressed people.

### Computerised interventions

#### Description and rationale

Computerised interventions consist of the presentation of information via the internet or computerised cognitive behaviour therapy (CBT), which is the provision of structured sessions of CBT via computer. The delivery method can be over the internet or via interactive CD-ROM, and the level of professional involvement can vary from none to substantial. Although some computerised CBT packages are only available through a health professional, there are some which are freely available on the internet [87-90].

#### Review of efficacy

##### Depressive disorders

A meta-analysis of 5 RCTs examined the effects of internet-based CBT on depression over weeks or months in a total of 1,982 adults recruited from a mix of clinical and community sources [91]. The meta-analysis showed an overall small difference in depression between the internet CBT and control groups (fixed effects analysis d = 0.27, 95% CI 0.15 to 0.40; mixed effects analysis d = 0.32, 95% CI 0.08 to 0.57). The trials were of reasonable to good quality and had no professional involvement in four. Similarly, another review of eight RCTs found that computerised CBT without professional involvement had a small effect on depression, but that computerised CBT with professional involvement had a bigger effect, similar to that achieved in face to face CBT [92]. The author proposed that the smaller effect on depression of unsupervised computerised CBT could be due to low completion rates caused by the absence of a motivating professional. Only one RCT has examined the use of a depression information website [93]. This intervention was found to produce significantly greater change in depression than a control condition and was not significantly different from web-based CBT.

##### Non-clinically depressed

A controlled trial in 59 adolescent males of MoodGYM, an internet-based CBT program, compared 5 weekly sessions of in-class use of MoodGYM with the usual personal development class scheduled at that time [94]. There was no significant difference in change in depression symptoms between the two groups. However, compliance was low, with only 40% completing at least half of the MoodGYM program.

#### Conclusion

The evidence for computerised interventions for depressive disorders appears promising, particularly if a professional is involved. Pure self-help computerised CBT is not as helpful, but is a potentially beneficial option for those who are sufficiently motivated to complete the program on their own. There is insufficient evidence to determine the helpfulness of computerised interventions in those without depressive disorders.

### Distraction

#### Description and rationale

Distraction is directing attention away from the symptoms of depression and its possible causes and consequences and towards pleasant or neutral thoughts and actions. Response styles theory [95] proposes that rumination in response to depressed mood worsens and prolongs it, whereas distraction reduces the intensity and duration of depressed mood. Depressed people tend to ruminate on their depression and depressed mood, in the belief that this will lead to greater understanding and better problem solving. However, ruminating whilst in a depressed mood is likely to lead to more negative thinking and make depression symptoms seem more prominent. Distraction may interfere with rumination and its distortions in thinking and allow better problem solving once the depressed mood has improved.

#### Review of efficacy

##### Depressive disorders

A number of experiments have been conducted on the effects of distraction on depressed mood over minutes, in both clinically depressed people and people with a high score on a depression symptom scale [96-103]. A number of distraction tasks have been used, such as thinking about and visualising a series of neutral external things (for example, the shape of the African continent or the layout of a typical classroom), describing pictures, playing a board game, or thinking about broad social issues. Many of these experiments have compared distraction to a rumination task which involves focusing on current feelings and personal characteristics, such as 'your feelings right now and why you are feeling this way'. The generally consistent finding has been that rumination increases or maintains depressed mood, whereas distraction reduces depressed mood. The few studies that compared distraction with a control (such as sitting quietly or receiving no instructions from experimenters) also show that distraction is better at reducing depressed mood [104-107].

##### Non-clinically depressed

Other studies have experimentally induced depressed mood in non-clinically depressed participants before applying distraction [107-113] and have also typically found that distraction reduces depressed mood.

#### Conclusion

There is good evidence that distraction (in the form of thinking or visualising pleasant or neutral thoughts) is helpful for temporarily alleviating depressed mood, particularly if the alternative is ruminating on the causes and consequences of it. Other strategies may be required once the mood has lifted to prevent the recurrence of depressed mood.

### Meditation

#### Description and rationale

Meditation refers to a variety of self-regulation practices that focus on training attention and awareness. Different forms may emphasise concentration on something (such as an inner sound or the breath) as in transcendental meditation, or awareness of thoughts without judgement, as in mindfulness meditation or vipassana. Although meditation is often undertaken to achieve spiritual or religious goals, this is not a requirement of practice, and it has even been combined with Western treatments, such as mindfulness-based stress reduction, and mindfulness-based cognitive therapy. Meditation aims to reduce anxiety and promote relaxation. Additionally, mindfulness meditation may be helpful for depression because it leads to a distancing of self from negative thoughts and reduces rumination.

#### Review of efficacy

##### Non-clinically depressed

Five RCTs have evaluated the effects of meditation on depressed mood or symptoms in non-clinically depressed individuals, with inconsistent results. An RCT in 73 elderly of transcendental meditation versus other mental relaxation or concentration tasks or waitlist found no significant difference in depression between groups after 12 weeks [114]. An RCT in 42 young adults that compared mindfulness meditation with guided visual imagery for 3 weeks found that neither intervention had an effect on depressed mood [115]. In contrast to these two trials, three RCTs found an effect. An RCT in 150 adults who participated in a week-long Buddhist meditation retreat found that the meditation group had significantly reduced depression symptoms compared with the delayed treatment control group [116]. An RCT in 61 adults who were assigned to 1 of 2 meditation groups or a control group, found that those assigned to the group using an Indian Vedic mantra (hypothesised to be particularly helpful for depression) had a significantly greater reduction in depression symptoms after 28 days of meditating, compared with either the control group or the group using a mantra composed of meaningless Sanskrit syllables [117]. Finally, an RCT that induced a depressed mood in 177 young adults found that a short mindfulness meditation significantly improved mood more than a distraction or rumination task [112].

#### Conclusion

For non-clinically depressed individuals, the evidence for meditation is inconsistent, with some trials showing benefit and others not. There is no evidence on the effects of meditation on depressive disorders.

### Relaxation training

#### Description and rationale

This review concerns relaxation training based primarily on progressive muscle relaxation, which involves teaching a person to relax voluntarily by tensing and relaxing specific muscle groups. Relaxation training may be helpful for depression because it improves a person's ability to deal with anxiety, and anxiety may lead to depression.

#### Review of efficacy

##### Depressive disorders

Nine RCTs have evaluated progressive muscle relaxation training in adolescents or adults with depressive disorders [118-126]. The number of participants undergoing relaxation training varied between 8 and 43, the number of relaxation sessions varied from 5 to 40, and the training was delivered by trained persons in 7 of these trials. An unpublished meta-analysis of four of these trials [120,123,124,126] that compared relaxation training with wait-list or minimal treatment control groups found significantly lower depression scores overall after treatment in the relaxation group (d = -0.66, 95% CI -1.07 to -0.25). However, a meta-analysis of results from six trials [120-125] comparing relaxation with psychological treatment found that relaxation was significantly less helpful in reducing depression than psychological therapy (d = 0.52, 95% CI 0.25 to 0.79).

##### Non-clinically depressed

Five RCTs have compared progressive muscle relaxation training with a placebo or non-treatment control in non-depressed individuals [127-131]. All found that relaxation was no better than the control in reducing depression symptoms or depressed mood. These trials varied in the age of participants (from children to older adults), duration of the intervention (6 to 11 weeks), length of relaxation sessions (5 min to 1.5 h), and whether the training was administered in a group or by the participant at home.

#### Conclusion

Research suggests that progressive muscle relaxation training may be helpful for those with depressive disorders, although it may not be as helpful as psychological treatment. It does not appear beneficial for depression in non-clinically depressed individuals.

## Lifestyle changes

### Exercise

#### Description and rationale

The two main types of exercise are aerobic (exercises the heart and lungs, such as in jogging) or anaerobic (strengthens muscles, such as in weight training). The antidepressant mechanism is unclear. Proposed mechanisms include physiological factors, such as effects on sleep regulation or serotonin and endorphins. Proposed psychological mechanisms include the interruption of negative thoughts that may prolong or worsen depression, or an increase in perceived coping ability. Exercise is also incompatible with inactivity and withdrawal, which are common unhelpful coping strategies for depression [132].

#### Review of efficacy

##### Depressive disorders

The most recently published meta-analysis of exercise for depression restricted included trials to those with adults or older adults who were clinically depressed [132]. Results were pooled from 11 RCTs involving 513 participants that compared exercise to a control condition (waitlist, placebo, low-intensity exercise or health education). Exercise interventions varied in frequency from between two to four times weekly, in duration between 20 and 45 min, and in intensity between unspecified and 70–85% maximum heart rate, for up to 12 weeks. The meta-analysis found an overall very large difference in depression between the two groups, with exercise being more effective (d = 1.42, 95% CI 0.92 to 1.93). Preliminary subgroup analyses indicated that anaerobic exercise may be as effective as aerobic exercise. A systematic review examining exercise specifically in older adults found 5 RCTs involving 318 older adults with depression (diagnosed or high level of symptoms), varying between 6 and 16 weeks in duration [133]. Compared to controls, depression symptoms were significantly lower in the exercise condition (both aerobic and anaerobic) in four of the five trials, although trials were not of high quality. Exercise has also been systematically reviewed as an intervention in children and young people with depressive disorders [134]. Three small trials were found, involving 81 participants. These were of low to moderate quality and all indicated no significant difference in outcome between exercise and various control conditions. The authors concluded that the evidence base was too scarce to determine the effect of exercise on depression in children and young people.

##### Non-clinically depressed

A non-systematic review of trials evaluating the effect on depression symptoms of aerobic exercise in non-depressed adults found that older lower quality studies had mixed results, but more recent RCTs generally find no reduction in depression symptoms [135]. A systematic review of exercise for depression in non-depressed older adults found 5 RCTs involving 766 participants [133]. Trials lasted between 12 weeks and 12 months. Three trials comparing aerobic exercise with control interventions had mixed results, whereas two trials comparing anaerobic exercise with controls found no significant difference in reduction of depression symptoms. A systematic review of exercise for depression in non-depressed young people found exercise more effective than no intervention in 5 low quality trials involving 145 participants [134]. Conversely, there was no effect of exercise found in 2 low quality trials comparing exercise with low intensity exercise (182 participants) and 2 low to moderate quality trials comparing it to psychosocial interventions (161 participants). Researchers have also investigated the effects over minutes of a single session of exercise on depressed mood. A selective review found 17 trials in non-depressed adolescents or adults where a variety of exercise (aerobic dance, yoga, jogging, rock climbing, swimming, tai chi and walking), ranging in duration from 10 to 80 min, had made improvements to depressed mood in participants [136]. The review did not indicate whether trials were controlled. The authors noted that positive effects depend upon complex interactions between participant characteristics (such as whether they found the exercise enjoyable), exercise mode (such as whether it is competitive or non-competitive), and exercise practice conditions (such as intensity and duration).

#### Conclusion

There is good evidence that exercise is helpful for reducing depression symptoms in adults with depressive disorders. It also appears to be helpful for older adults with depressive disorders; however there is insufficient evidence to determine the helpfulness in children and young people. Results from studies in non-clinically depressed individuals are mixed, perhaps reflecting the reduced room for improvement in these individuals. However, there is some evidence that single sessions of exercise may improve depressed mood in non-clinically depressed individuals. Research has yet to clarify the most appropriate dose and type of exercise required for an effect.

### Humour

#### Description and rationale

Laughter has similar physiological effects as vigorous exercise, such as reducing stress hormones, relieving tension, and releasing endorphins into the brain [137,138]. Responding to a stressful situation with humour may also help depression by causing a shift in thinking, promoting objectivity and distance from the threat or problem [137,138].

#### Review of efficacy

##### Non-clinically depressed

Two RCTs have examined humour's effect on depressed mood after exposure to stress or a negative mood induction. A trial with 38 young adults who underwent a depressed mood induction found that only listening to a humorous tape restored mood to pre-experimental levels, compared with a neutral tape or no tape [139]. An RCT in 80 males found that those who produced a humorous narration instead of a serious narration to a stressful silent film had significantly lower levels of depressed mood, although the effect did not last beyond 15 min [138]. The only trial to evaluate effects lasting longer than minutes was an RCT in 61 nursing home residents that examined the effect of humorous weekly group sing-a-longs on depression symptoms [140]. Compared to residents in control homes who received no intervention, those in the sing-a-long groups had significantly reduced depression symptoms after 4 weeks on one measure, but not on another. However, it is not clear whether the humour or other aspects of the intervention (such as social interaction or singing) were responsible for the effect.

#### Conclusion

Limited evidence suggests that exposure to humour (such as by watching a humorous video) temporarily improves depressed mood. Longer-term effects have not been adequately investigated. There is no evidence on the effects of humour in depressive disorders.

### Pets

#### Description and rationale

Spending time with pets might improve relaxation levels in their owners, provide companionship and a buffer against loneliness, and strengthen a sense of responsibility and self respect.

#### Review of efficacy

##### Non-clinically depressed

Two RCTs have examined the effects of live-in birds assigned individually to older adults. Half of 40 older adult residents of skilled rehabilitation units received a caged budgerigar in their room for 10 days [141]. The group who received a bird had significantly reduced depression scores at the end of the trial, although the authors noted this result might have been caused by an increase in human visitors to see the bird. A larger, better designed trial also had a similar result. A total of 144 nursing home residents were given a canary, a plant or nothing to look after in their rooms for 3 months [142]. Depression symptoms significantly improved in the group assigned canaries, but not in the other two groups.

#### Conclusion

Studies in non-clinically depressed elderly nursing home residents suggest a positive effect of live-in pets on depression symptoms. These results may not generalise to the broader population. There is no evidence on the effects of pets on depressive disorders.

### Pleasant activities

#### Description and rationale

Depressed people engage in pleasant activities less often and find fewer activities pleasant compared with other people. Increasing engagement in pleasant activities can be performed informally or included as part of activity scheduling in cognitive behaviour therapy (CBT). Increasing the frequency of pleasant activities is thought to improve depressed mood by increasing opportunities for the reinforcement of healthy (non-depressed) behaviour and countering avoidance, withdrawal and inactivity.

#### Review of efficacy

##### Depressive disorders

A meta-analysis of RCTs of activity scheduling for depression in adults found clear indications that it is effective [143]. A total of 10 studies compared activity scheduling with a control (usually a delayed treatment) and there was an overall large difference in depression, favouring activity scheduling (d = 0.87, 95% CI 0.60 to 1.15). A total of 14 studies compared activity scheduling with other psychological treatments (usually cognitive therapy), and overall there was no difference in depression after treatment (d = 0.13, 95% CI -0.05 to 0.30). Trials were generally small and not of the highest quality. However, these trials only examined activity scheduling as a treatment from a professional. Other factors, such as the therapeutic relationship, ritual of the therapy, or even other treatment components in particular trials, such as social skills training, may play a role in treatment outcome. Therefore, it is difficult to generalise these findings to a self-help method of activity scheduling.

##### Non-clinically depressed

An RCT of 65 non-depressed young adults had 3 groups: a monitor only control group, who monitored their daily activities and mood; a behaviour group, who additionally increased the number of activities found pleasurable; and a cognitive/behaviour group, who in addition to increasing pleasurable activities, focused on the positive aspects of the pleasant activities and the benefits of participating in them [144]. After 2 weeks, depression scores significantly decreased for the monitor group and the cognitive/behaviour group, but not for the behaviour group. This finding was interpreted as support for the view that cognitive processing is required in addition to activity scheduling for an antidepressant effect, but this does not account for the decrease in depression shown in the monitor group.

#### Conclusion

There is reasonably good evidence that professional treatment involving activity scheduling is helpful for depression. This effect may not be applicable to a depressed person who independently attempts to increase pleasant activities. The evidence for the helpfulness of activity scheduling is inconclusive in non-clinically depressed individuals.

### Prayer

#### Description and rationale

Prayer has traditionally been used in times of illness and is often used by the public to help cope with mental health problems.

#### Review of efficacy

##### Non-clinically depressed

One RCT in 88 Christian adults found practicing the Jesus prayer ('Lord Jesus Christ, have mercy on me') for 10 min daily for 30 days lowered depression scores significantly more than a non-treatment control group [145].

#### Conclusion

Limited evidence suggests prayer may be helpful for depressive symptoms in Christians who are not clinically depressed. There is no evidence on the effects of prayer on depressive disorders.

### Qigong

#### Description and rationale

Qigong is a 3,000-year-old Chinese self-training method involving meditation, breathing exercises and body movements. Qigong regulates the flow of qi (energy) throughout the body, removing imbalances or blockages, which cause emotional disturbances or physical symptoms.

#### Review of efficacy

##### Non-clinically depressed

One crossover trial with order randomised has evaluated the effects over minutes of qigong on depressed mood [146]. A total of 15 older adults recruited from existing qigong classes participated in a session of both qigong and brisk walking. Level of depressed mood did not significantly change after either session.

#### Conclusion

There is insufficient evidence to determine whether qigong is helpful for depressed mood in non-clinically depressed individuals. There is no evidence on the effects of qigong on depressive disorders.

### Sleep deprivation

#### Description and rationale

Total sleep deprivation is staying awake for a whole night and the following day, without napping. Partial sleep deprivation is restricting sleep to either the early or latter part of the night and remaining awake for the remainder of the night. Although the antidepressant mechanism is poorly understood, many have been proposed, such as normalisation of metabolic activity within the limbic system, or effects on serotonin functioning [147].

#### Review of efficacy

##### Depressive disorders

Reviews of the efficacy of sleep deprivation for depressive disorders conclude that about 60% of depressed individuals improve after sleep deprivation [147,148]. The degree of symptom change ranges from complete remission to worsening in a minority. The effect is delayed in some individuals who only respond following sleep the next day. The evidence is unclear, but partial sleep deprivation may be as effective as total sleep deprivation [147]. Although the antidepressant effect is rapid, it typically does not last, with 50–80% of responders suffering a complete or partial relapse following recovery sleep. Researchers have attempted to prevent relapse with other treatments such as antidepressant drugs, shifting of sleep time or light therapy, which show promise for reducing the risk of relapse.

##### Non-clinically depressed

One RCT in 40 males found a significant increase in depressed mood after 24 h of wakefulness as compared to controls who had a typical night's sleep [149].

#### Conclusion

Evidence is consistent that sleep deprivation is helpful for many individuals with depressive disorders, although the effects are typically temporary. In non-clinically depressed individuals, sleep deprivation may cause an increase in depressed mood.

### Tai Chi

#### Description and rationale

Tai chi is a type of moving meditation that originated in China as a martial art. It involves slow, purposeful movements and focused breathing and attention. In traditional Chinese medicine, tai chi is thought to benefit health through the effects of stereotyped hand and foot movements on important acupoints and visceral channels [150]. Tai chi could affect depression because it is a form of moderate exercise or because it is a relaxing distraction from anxiety and stress.

#### Review of efficacy

##### Non-clinically depressed

Two RCTs have compared tai chi with different forms of exercise or relaxation. A total of 96 healthy adult tai chi practitioners participated in 1 h of tai chi, brisk walking, tai chi meditation or neutral reading after being subject to experimentally induced emotional or mental stress [151]. All activities significantly improved depressed mood. Another trial compared a 16-week program of tai chi against low or moderate intensity walking, low intensity walking with relaxation, and no treatment in 135 adults [152]. Depressed mood significantly improved in women in the tai chi group compared to those in the control, but changes in depressed mood in men did not differ significantly between the different groups.

#### Conclusion

There is insufficient evidence to determine whether tai chi is helpful for depressed mood in non-clinically depressed individuals. There is no evidence on the effects of tai chi on depressive disorders.

### Yoga

#### Description and rationale

Yoga exercises the mind and body through physical postures, breathing techniques and meditation. Each posture is held for a period of time and synchronised with breathing. Yoga is thought to relieve stress and improve relaxation, but it may also be effective due to feelings of mastery from learning difficult postures, or improvements in body image from greater bodily awareness and control.

#### Review of efficacy

##### Depressive disorders

A recent systematic review of randomised controlled trials of yoga for depression found five trials to review [153]. The studies varied in the type of yoga studied (such as Iyengar, Shavasana, and Sudarshan Kriya Yoga), severity of depression (mild to severe), number of participants per yoga group (10–25), and length of intervention (3 days to 5 weeks), and participants were all under 50 years. Nevertheless, the authors concluded that yoga for depressive disorders is potentially beneficial, but that further investigation is needed.

##### Non-clinically depressed

Three RCTs have evaluated the effects over months of yoga classes on depressive symptoms or depressed mood, with inconsistent results. Two trials in older adults of 60–90 min yoga sessions once or twice a week for 16 weeks to 6 months found no effect on depressed mood or symptoms relative to controls [154,155]. However one shorter trial of 6 weeks in adults found depressed mood significantly improved in the yoga group compared with a wait-list control group [156].

#### Conclusion

Initial evidence suggests that yoga may be beneficial for depressive disorders. The evidence is inconsistent for effects in non-clinically depressed individuals.

## Physical and sensory methods

### Aromatherapy

#### Description and rationale

Aromatherapy is the therapeutic use of essential oils, which are highly concentrated extracts of plants. Essential oils can be diluted in carrier oils and absorbed through the skin via massage, or heated and vaporised into the air. Essential oils said to have antidepressant effects include bergamot, geranium, jasmine, lavender and Egyptian rose [157]. They are available from health food shops or pharmacies. The antidepressant mechanism is unclear, but may be due to the odour either being perceived as pleasant or triggering memories and emotions that affect mood. Alternatively, the oil's chemical constituents may be absorbed into the blood stream and have pharmacological effects [157].

#### Review of efficacy

##### Non-clinically depressed

One RCT has examined aromatherapy's effects over minutes on depressed mood in non-clinically depressed adults. A total of 73 adults were exposed to water or essential oils of lavender or rosemary for 10 min whilst they completed a stressful mental task. At the end of the task there was no significant difference in depressed mood between groups [158].

#### Conclusion

There is insufficient evidence to determine whether aromatherapy is helpful for depressed mood in non-clinically depressed individuals. There is no evidence on the effects of aromatherapy in depressive disorders.

### Hydrotherapy

#### Description and rationale

Hydrotherapy includes hot air and steam baths or saunas, wet packings, and various kinds of warm and cold baths [159]. Hydrotherapy was a popular historical treatment for depression and was thought to promote relaxation [159].

#### Review of efficacy

##### Non-clinically depressed

An RCT in 40 adults found no effect on depressed mood of a 10-min immersion in a spa bath with either the whirlpool motor on or off [160].

#### Conclusion

Limited evidence suggests that hydrotherapy is not effective for the relief of depression symptoms or depressed mood. There is no evidence on the effects of hydrotherapy on depressive disorders.

### Light therapy

#### Description and rationale

Light therapy is exposure of the eyes to bright light for an appropriate duration, often in the morning. The light is emitted from a box or lamp which the person sits in front of. Several manufacturers make their own versions of light therapy devices, some of which have not been evaluated in clinical trials. These can be bought over the internet. Different devices may use different parts of the light spectrum, at different intensities of illumination. Light therapy was originally used to treat seasonal affective disorder (SAD), by advancing the phase delay in circadian rhythms caused by exposure to less sunlight in winter. It has now been extended to treat non-seasonal depression and therefore the phase advance is probably not the only mechanism [161].

#### Review of efficacy

##### Depressive disorders

Two recent meta-analyses have been carried out. The first examined light therapy for depression and SAD in non-geriatric adults over days or weeks [162]. Included studies were RCTs of a reasonably high standard, with light therapy groups receiving adequate doses of light exposure. Three studies were included in the meta-analysis of light therapy for depression, eight for light therapy for SAD, and five for dawn simulation for SAD. The meta-analyses revealed a large effect of light therapy (d = 0.84, 95% CI 0.60 to 1.08) and dawn simulation on SAD (d = 0.73, 95% CI 0.37 to 1.08), compared with placebo, and a medium-sized effect of light therapy on depression (d = 0.53, 95% CI 0.18 to 0.89). Another meta-analysis looked exclusively at light therapy for non-seasonal depression and applied broader inclusion criteria for trials [163]. A total of 18 RCTs were analysed, however only 2 used light therapy as the only treatment. Pooling results from these two studies showed that light therapy was beneficial when using a fixed effects analysis, but the result did not reach significance using a random effects model. A systematic review of light therapy for depressed children and adolescents also concluded that limited evidence suggests it is helpful for winter depression, but not for non-seasonal depression [19].

##### Non-clinically depressed

Four RCTs have evaluated light therapy in non-clinically depressed individuals over days or weeks, with mixed findings. Trials with participants who experienced winter difficulties (subsyndromal SAD) found light therapy helpful for depressed mood or symptoms [164,165]. Light therapy doses were 2,500 lux for 2–5 h in the morning or split over both morning and evening, Trials with participants who had no winter difficulties generally did not find light therapy helpful [164-167] and some of these participants experienced negative effects, such as irritability, after light therapy [166].

#### Conclusion

There is good evidence that light therapy is effective for SAD (winter depression). It also appears to be helpful for non-seasonal depressive disorder, but the evidence is not as strong and the effect is smaller. It may also be helpful for non-clinically depressed individuals who experience mild symptoms of SAD.

### Massage

#### Description and rationale

Massage is thought to work by stimulating vagal activity, leading to a reduction of stress hormones and physiological arousal; or by influencing body chemistry, such as increasing serotonin or releasing endorphins [168].

#### Review of efficacy

##### Depressive disorders

Two RCTs have evaluated the effects over minutes of massage in people with depressive disorders. One trial was of a 30-min massage in children or adolescents with dysthymia [169], and one was of a 20-min massage in depressed pregnant women [118]. Both trials compared massage with a form of relaxation and found massage significantly reduced depressed mood compared with relaxation. These trials also evaluated the effects of multiple doses of massage. Changes in depressed mood or symptoms were examined over the duration of each trial (lasting 5 days and 16 weeks respectively), with both finding a significant reduction in the massage group compared with controls.

##### Non-clinically depressed

Four RCTs have evaluated the effects over minutes of massage on non-depressed adults compared to some control intervention. The results have been variable. One RCT found massage produced greater effects than reading [170], however two RCTs found no significant difference from relaxation [171,172] and one found no difference from resting [173]. Massages were restricted to the upper body and were 10 to 30 min long. One RCT has evaluated multiple doses of massage, but only against relaxation therapy. A total of 50 adults were given a 15-min massage or were told to tighten and relax their muscles twice a week for 5 weeks. Depressed mood in both the massage group and the relaxation control group significantly improved [171].

#### Conclusion

Preliminary evidence suggests massage may have immediate and longer-term effects on depressed mood and symptoms in those with depressive disorders. The evidence for immediate and longer-term effects of massage in those who are not depressed is inconsistent. It should be noted that in virtually all studies massage was given by trained massage therapists, and the effects of massage given by self or non-trained professionals has not been evaluated.

### Music

#### Description and rationale

Music has been called the 'language of emotions' and appears to activate emotional systems in the brain. It is unclear to what degree the emotional impact is caused by specific attributes of the music itself (such as rhythm and melody), or cultural context and memories [174].

#### Review of efficacy

##### Non-clinically depressed

Listening to music (both classical and modern) has frequently been used in experimental settings to induce particular moods in participants. A meta-analysis of these studies concluded that music is a moderately effective method of inducing temporary depressed or elated mood in experimental settings with non-depressed individuals [175]. Only one RCT has examined the effects of listening to music over weeks [176]. A total of 102 adult female nurses participated in a 6-week trial where they were instructed to listen to 20 min of music self-selected to be stress reducing three times a week, perform 20 min of self-selected aerobic exercise three times a week or maintain their usual exercise and stress-reduction activities. Listening to music did not reduce depression symptoms significantly more than the control group.

#### Conclusion

Listening to music can be an effective method of lifting mood in the short term (less than an hour) in non-clinically depressed individuals, but there is no evidence that music can reduce depression over periods of days or weeks. There is no evidence on the effects of music on depressive disorders.

### Negative air ionisation

#### Description and rationale

A negative air ioniser is a device that uses high voltage to electrically charge air particles. The antidepressant mechanism is unknown but may involve effects on both central and peripheral serotonergic activity [177].

#### Review of efficacy

##### Depressive disorders

One RCT in participants with seasonal affective disorder found significantly greater reduction in depression symptoms over weeks with 30 min daily exposure to high density negative ionisation (4.5 × 10^13^/s flow rate) compared with a placebo of low density negative ionisation [178].

##### Non-clinically depressed

One RCT [167] compared exposure to high density (4.5 × 10^14^/s) negative ion generators, bright light, music or low density (1.7 × 10^11^/s) negative ion generators (placebo) for 30 min in 118 non-depressed adults. The high density negative ions significantly decreased depressed mood compared with placebo. Another study of depressed mood in non-depressed adults found that exposure to high concentrations of negative ions significantly decreased depressed mood compared with exposure to low concentrations of negative ions. However, depressed mood was significantly increased in those who were experimentally manipulated to feel angry and exposed to high negative ion concentrations, compared with those who were also experimentally manipulated to feel angry but were exposed to low negative ion concentrations [179].

#### Conclusion

A small number of studies suggest exposure to high density negative ions (at least 2.7 × 10^6^/cm^3^) is helpful for seasonal affective disorder and depressed mood in non-clinically depressed individuals.

### Singing

#### Description and rationale

Music elicits strong emotional responses in humans, however singing may also improve mood through changes in breathing patterns, the expression of emotion, or through the content of lyrics [180].

#### Review of efficacy

##### Non-clinically depressed

Effects over weeks were examined in an RCT involving 61 nursing home residents who participated in humorous weekly group sing-a-longs [140]. Compared to residents in control homes who received no intervention, those in the sing-a-long groups had significantly reduced depression symptoms after 4 weeks on one measure, but not on another. However, it is not clear whether the singing or other aspects of the intervention (such as humour or social interaction) were responsible for the effect.

#### Conclusion

Limited research suggests that group singing may improve depressed mood or depression symptoms in non-clinically depressed individuals, however these results require replication. The effects of singing have not been examined in individuals with depressive disorders.

## Discussion

The self-help interventions with the best evidence of efficacy for depressive disorders are S-adenosylmethionine, St John's wort, bibliotherapy, computerised interventions, distraction, relaxation training, exercise, pleasant activities, sleep deprivation, and light therapy. With the possible exception of St John's wort, these interventions have been less researched than standard treatments provided by a professional such as antidepressants or cognitive behaviour therapy. Preliminary evidence also appears promising for a number of other interventions; however these have received less research attention. These include borage, carnitine/acetyl-L-carnitine, saffron, autogenic training, yoga, massage, and negative air ionisation.

There were fewer interventions with good or preliminary evidence in non-clinically depressed samples. Promising interventions with immediate effects on depressed mood include distraction, exercise, humour, music, negative air ionisation, and singing. Autogenic training, light therapy, omega 3 fatty acids, pets, and prayer may have helpful longer-term effects over days or weeks. The mechanism of action for many interventions is unclear, and for some with promising effects the mechanism is completely unknown, for example, negative air ionisation. Studies in non-clinically depressed samples may include participants with varying degrees of depressive symptoms, from none through to symptoms at the threshold for major depression. The context behind depressive symptoms is also unknown: symptoms could be residual following resolution of a major depressive episode, prodromal to a major depressive episode, ongoing, or reactions to life stresses or bereavement. As such, it is not surprising that fewer studies had positive results than studies in participants with depressive disorders, even though it is probable that some self-help interventions are effective in reducing depressive symptoms within specific ranges of symptom severity and in particular contexts. Another problem is measuring change in symptoms in populations near the normal end of the depression spectrum. A lack of instrument sensitivity to small changes in symptoms may be responsible for no significant changes detected in many trials with non-clinically depressed participants. As prodromal symptoms of depressive disorders appear to involve anxiety and irritability [181], it may be more appropriate to measure general psychological distress in these populations with lower levels of depressive symptoms, for example by using instruments such as the K10 questionnaire [182].

Although some interventions appear promising, there remains much to be learned about active ingredients and mechanisms, the specification of activities, behaviours and intervention content (for example, for exercise, the type and ideal dosage), as well as possible side effects and safety issues. Also, interventions were conducted in ideal conditions with at least some degree of professional involvement. Whether these effects generalise to conditions of informal self-help, where there is no professional involvement, is yet to be evaluated. Many of the trials were poor quality, suffering from short durations with no follow-up, little information on attrition, possible blinding issues, or had yet to be replicated by other research groups. Differential effects across age groups have not received much attention either.

For the majority of interventions searched, there were no trials available to review, and for some interventions there was a lack of research on their use as monotherapy. Many self-help strategies for depressive symptoms are not feasible or ethical to evaluate in RCTs, such as taking time off work, and may require alternative approaches to evaluating evidence. One approach is to ask individuals who have experienced depression what they find personally helpful [183]. This approach found that exercise, yoga/meditation, massage, and relaxation were rated highly and as strongly as professionally recommended strategies such as CBT and SSRIs. Another approach is to develop consensus of experts on what works best. We are currently undertaking just such a project using the Delphi [184,185] method of consensus, by gathering the views of expert clinicians and consumers on what self-help strategies are likely to be most helpful for subthreshold depressive symptoms.

Some interventions with good or reasonable evidence are very feasible to implement by an individual and would fit well into a promotional campaign. Others may be less feasible due to the need to purchase expensive equipment or supplements, or require an investment in time or effort to learn. As there may be no support or monitoring from professionals, the risk/benefit ratio would need to be low as well.

There are a number of limitations to this review, including a search restricted to articles written in English; restricting the reporting of mood effects to that of depressed mood only, rather than including other possible relevant mood variables such as energy level, fatigue or anxiety; and the number of interventions reviewed which precluded a more detailed analysis of each intervention.

## Conclusion

A number of self-help interventions have promising evidence for reducing subthreshold depressive symptoms, although a larger evidence base is needed. Promotion of effective self-help strategies for subthreshold depressive symptoms could fit within a clinical staging model for depressive disorders. A clinical staging model allows for different intervention approaches at different stages of illness development. Intervening early during prodromal or subthreshold symptoms with benign but effective techniques could delay or prevent onset of depressive illness, reduce functional impairment, and prevent progression to other undesirable outcomes such as harmful use of substances [186]. The present review has identified a number of self-help interventions that could usefully be evaluated for prevention and early intervention with depressive symptoms.

## Competing interests

The authors declare that they have no competing interests.

## Authors' contributions

AM contributed to aspects of study design, carried out the literature searches, and drafted the reviews and manuscript. AJ conceived the study, contributed to the reviews and helped to draft the manuscript. Both authors read and approved the final manuscript.

## Supplementary Material

Additional file 1 **Search strategy**. Microsoft Word document of literature search strategy used. Click here for file
