# Supplementary material for: Self-help interventions for depressive disorders and depressive symptoms: a systematic review
Source: Ann Gen Psychiatry. 2008 Aug 19;7:13. doi: 10.1186/1744-859X-7-13 (PMC2542367; doi:10.1186/1744-859X-7-13)
Supplement: Additional file 1 — Search strategy. Microsoft Word document of literature search strategy used. [file 1744-859X-7-13-S1.doc]

## Electronic searching

Database: CSA Illumina PsycINFO from database inception to present; NLM PubMed from database inception to present; Wiley InterScience Cochrane Database of Systematic Reviews from database inception to present

Format of search: Search-terms AND (Depressi* OR Dysthym* OR Affective OR Mood)

| **Treatment** | **Search terms** | **Date** | **Number of hits** |
| --- | --- | --- | --- |
| Yoga | yoga^a^ | 22/12/2006 | 362 |
|  | yoga^b^ | 8/01/2007 | 65 |
|  | yoga[[1]](#footnote-2)§c | 17/01/2007 | 4 |
| Light therapy | light therapy OR phototherapy^§c^ | 6/03/2007 | 32 |
|  | light therapy[TIAB] OR phototherapy[TIAB][[2]](#footnote-3)†b | 6/03/2007 | 97 |
|  | light therapy[TIAB] OR phototherapy[TIAB] AND (non-clinical OR normal OR subsyndromal)^b^ | 9/08/2007 | 31 |
|  | KW=(light therapy OR phototherapy)[[3]](#footnote-4)†a | 6/03/2007 | 41 |
|  | KW=(light therapy OR phototherapy) AND (non-clinical OR normal OR subsyndromal)^a^ | 9/08/2007 | 98 |
| Selenium | selenium^a^ | 4/01/2007 | 69 |
|  | selenium^b^ | 8/01/2007 | 47 |
|  | Selenium^§c§^ | 17/01/2007 | 11 |
| Tyrosine | KW=tyrosine^a^ | 5/01/2007 | 205 |
|  | tyrosine[TIAB]^b^ | 8/01/2007 | 342 |
|  | tyrosine^§c^ | 17/01/2007 | 1 |
| Tai-chi | tai chi OR t'ai chi^a^ | 5/01/2007 | 133 |
|  | tai chi OR t'ai chi^b^ | 8/01/2007 | 17 |
|  | tai chi OR t'ai chi^§c^ | 17/01/2007 | 2 |
| Aromatherapy | aromatherapy OR essential oil^a^ | 8/01/2007 | 127 |
|  | aromatherapy OR essential oil^b^ | 8/01/2007 | 44 |
|  | aromatherapy OR essential oil^§c^ | 17/01/2007 | 9 |
| Fragrance | KW=(fragrance OR perfume OR cologne OR scent OR odor OR odour)^a^ | 27/09/2007 | 140 |
|  | fragrance OR perfume OR cologne OR scent^b^ | 27/09/2007 | 97 |
|  | odour[TIAB] OR odor[TIAB]^b^ | 7/03/2007 | 132 |
|  | odour OR odor OR olfactory^§c^ | 7/03/2007 | 4 |
| Chocolate | KW=chocolate^a^ | 8/01/2007 | 71 |
|  | chocolate^b^ | 8/01/2007 | 37 |
|  | chocolate^§c^ | 17/01/2007 | 0 |
| Ginkgo biloba | ginkgo biloba^a^ | 9/01/2007 | 210 |
|  | ginkgo biloba^b^ | 9/01/2007 | 55 |
|  | ginkgo biloba^§c^ | 17/01/2007 | 6 |
| Lemon balm | lemon balm OR Melissa officinalis^a^ | 9/01/2007 | 21 |
|  | lemon balm OR Melissa officinalis^b^ | 9/01/2007 | 5 |
|  | lemon balm OR Melissa officinalis^c^ | 17/01/2007 | 3 |
| Glutamine | KW=glutamine^a^ | 9/01/2007 | 67 |
|  | glutamine^b^ | 9/01/2007 | 97 |
|  | glutamine^c^ | 17/01/2007 | 22 |
| Vervain | vervain OR Verbena^a^ | 9/01/2007 | 1 |
|  | vervain OR Verbena^b^ | 9/01/2007 | 0 |
|  | vervain OR Verbena^c^ | 17/01/2007 | 0 |
| Pets | pets^a^ | 9/01/2007 | 221 |
|  | pets^b^ | 9/01/2007 | 48 |
|  | pets^c^ | 17/01/2007 | 48 |
| Prayer | KW=prayer^a^ | 10/01/2007 | 184 |
|  | prayer [TIAB]^b^ | 10/01/2007 | 52 |
|  | prayer^c^ | 17/01/2007 | 7 |
| Music | DE=music^a^ | 11/01/2007 | 88 |
|  | music[TIAB]^b^ | 11/01/2007 | 305 |
|  | music^§c^ | 17/01/2007 | 13 |
| Omega-3 | KW=(omega-3 OR fish oil OR "essential fatty acid")^a^ | 11/01/2007 | 104 |
|  | omega-3 OR fish oil OR "essential fatty acid"^b^ | 11/01/2007 | 211 |
|  | omega-3 OR fish oil OR "essential fatty acid"^c^ | 17/01/2007 | 9 |
| Air ionisation | air ioni?ation OR negative ion^a^ | 15/01/2007 | 111 |
|  | air ionisation OR air ionization OR negative ion^b^ | 15/01/2007 | 21 |
|  | air ionisation OR air ionization OR negative ion^c^ | 17/01/2007 | 32 |
| Ginseng (chinese, american, siberian) | ginseng OR panax quinquefolius OR Eleutherococcus senticosus^a^ | 15/01/2007 | 105 |
|  | ginseng OR panax quinquefolius OR Eleutherococcus senticosus^b^ | 15/01/2007 | 38 |
|  | ginseng OR panax quinquefolius OR Eleutherococcus senticosus^c^ | 17/01/2007 | 22 |
| Sugar avoidance | KW=(sugar OR sucrose)^a^ | 15/01/2007 | 235 |
|  | sugar[TIAB] OR sucrose[TIAB]^b^ | 15/01/2007 | 198 |
|  | sugar OR sucrose^§c^ | 17/01/2007 | 31 |
| Natural progesterone | natural progesterone^a^ | 16/01/2007 | 13 |
|  | natural progesterone^b^ | 16/01/2007 | 9 |
|  | natural progesterone^c^ | 2/03/2007 | 57 |
|  | progestogens^a^ | 2/03/2007 | 68 |
|  | progestogens[TIAB]^b^ | 2/03/2007 | 31 |
| Dance or movement | KW=(dance OR movement therapy)^a^ | 16/01/2007 | 200 |
|  | dance OR movement therapy^b^ | 16/01/2007 | 51 |
|  | dance OR movement therapy^§c^ | 17/01/2007 | 60 |
| Vitamins | KW=(vitamin OR thiamine OR pyridoxine OR pyridoxamine OR cyanocobalamin OR ascorbic acid OR tocopherol OR tocotrienol OR biotin OR nicotinamide OR niacin OR nicotinic acid OR pantothenic acid OR pantothenate OR riboflavin OR folate OR folic acid OR naphthoquinone OR retinoids)^a^ | 17/01/2007 | 347 |
|  | vitamin OR thiamine OR pyridoxine OR pyridoxamine OR cyanocobalamin OR ascorbic acid OR tocopherol OR tocotrienol OR biotin OR nicotinamide OR niacin OR nicotinic acid OR pantothenic acid OR pantothenate OR riboflavin OR folate OR folic acid OR naphthoquinone OR retinoids^‡b^ | 17/01/2007 | 164 |
|  | vitamin^§c^ | 17/01/2007 | 83 |
|  | thiamine OR pyridoxine OR pyridoxamine OR cyanocobalamin OR ascorbic acid^c^ | 18/01/2007 | 88 |
|  | riboflavin OR folate OR folic acid OR naphthoquinone OR retinoids^§c^ | 18/01/2007 | 23 |
|  | nicotinamide OR niacin OR nicotinic acid OR pantothenic acid OR pantothenate^c^ | 18/01/2007 | 50 |
|  | tocopherol OR tocotrienol OR biotin^c^ | 18/01/2007 | 41 |
| S-Adenosylmethionine | S-Adenosylmethionine OR S-adenosyl methionine OR S-Adenosyl-L-Methionine OR SAM-e^a^ | 18/01/2007 | 196 |
|  | S-Adenosylmethionine OR S-adenosyl methionine OR S-Adenosyl-L-Methionine OR SAM-e^b^ | 18/01/2007 | 132 |
|  | S-Adenosylmethionine OR S-adenosyl methionine OR S-Adenosyl-L-Methionine OR SAM-e^c^ | 18/01/2007 | 13 |
| St John's wort | St John's wort OR St Johns wort OR hypericum perforatum^a^ | 19/01/2007 | 378 |
|  | St John's wort OR saint john's wort OR hypericum^b^ | 19/01/2007 | 338 |
|  | St John's wort OR hypericum^c^ | 19/01/2007 | 13 |
| Alcohol | DE=alcohol AND KW=((relax* OR treat*) OR (avoid* OR reduc* OR depriv*))^a^ | 23/01/2007 | 315 |
|  | alcohol drinking[MH] AND ((relax* OR treat*) OR (avoid* OR reduc* OR depriv*))^b^ | 23/01/2007 | 363 |
| Astragalus | astragalus OR astragalus membranaceus^a^ | 30/01/2007 | 2 |
|  | astragalus OR astragalus membranaceus^b^ | 30/01/2007 | 4 |
|  | astragalus^c^ | 30/01/2007 | 17 |
| Borage | borage OR Borago officinalis OR echium amoenum OR starflower^a^ | 30/01/2007 | 4 |
|  | borage OR Borago officinalis OR echium amoenum OR starflower^b^ | 30/01/2007 | 2 |
|  | borage OR Borago officinalis OR echium amoenum OR starflower^c^ | 30/01/2007 | 5 |
| Brahmi | brahmi OR Bacopa monniera OR Water Hyssop^a^ | 30/01/2007 | 9 |
|  | brahmi OR Bacopa monniera OR Water Hyssop^b^ | 30/01/2007 | 4 |
|  | brahmi OR Bacopa monniera OR Water Hyssop^c^ | 30/01/2007 | 0 |
| California poppy | California poppy OR Eschscholzia californica^a^ | 30/01/2007 | 0 |
|  | California poppy OR Eschscholzia californica^b^ | 30/01/2007 | 0 |
|  | California poppy OR Eschscholzia californica^c^ | 30/01/2007 | 0 |
| Catnip | catnip OR Nepeta cataria^a^ | 30/01/2007 | 4 |
|  | catnip OR Nepeta cataria^b^ | 30/01/2007 | 0 |
|  | catnip OR Nepeta cataria^c^ | 30/01/2007 | 0 |
| Cat's claw | cat's claw OR Uncaria tomentosa^a^ | 30/01/2007 | 2 |
|  | cat's claw OR Uncaria tomentosa^b^ | 30/01/2007 | 0 |
|  | cat's claw OR Uncaria tomentosa^c^ | 30/01/2007 | 0 |
| Gotu kola | Gotu Kola OR Asiatic Pennywort OR Antanan OR Pegaga OR Centella asiatica^a^ | 30/01/2007 | 4 |
|  | Gotu Kola OR Asiatic Pennywort OR Antanan OR Pegaga OR Centella asiatica^b^ | 30/01/2007 | 2 |
|  | Gotu Kola OR Asiatic Pennywort OR Antanan OR Pegaga OR Centella asiatica^c^ | 30/01/2007 | 3 |
| Cowslip | cowslip OR Primula veris^a^ | 31/01/2007 | 0 |
|  | cowslip OR Primula veris^b^ | 31/01/2007 | 0 |
|  | cowslip OR Primula veris^c^ | 31/01/2007 | 0 |
| Damiana | damiana OR Turnera diffusa OR Turnera aphrodisiaca^a^ | 31/01/2007 | 1 |
|  | damiana OR Turnera diffusa OR Turnera aphrodisiaca^b^ | 31/01/2007 | 2 |
|  | damiana OR Turnera diffusa OR Turnera aphrodisiaca^c^ | 31/01/2007 | 1 |
| Dandelion | dandelion OR Taraxacum officinale OR Taraxacum vulgare^a^ | 31/01/2007 | 5 |
|  | dandelion OR Taraxacum officinale OR Taraxacum vulgare^b^ | 31/01/2007 | 0 |
|  | dandelion OR Taraxacum officinale OR Taraxacum vulgare^c^ | 31/01/2007 | 2 |
| Flax seeds | flax seed OR flaxseed OR linseed OR Linum usitatissimum^a^ | 31/01/2007 | 3 |
|  | flax seed OR flaxseed OR linseed OR Linum usitatissimum^b^ | 31/01/2007 | 5 |
|  | flax seed OR flaxseed OR linseed OR Linum usitatissimum^c^ | 31/01/2007 | 9 |
| Ginger | ginger OR Zingiber officinale^a^ | 31/01/2007 | 68 |
|  | ginger OR Zingiber officinale^b^ | 31/01/2007 | 3 |
|  | ginger OR Zingiber officinale^c^ | 31/01/2007 | 0 |
| Hawthorn | KW=(hawthorn OR Crataegus laevigata OR Crataegus oxyacantha OR mayflower) ^a^ | 31/01/2007 | 17 |
|  | hawthorn OR Crataegus laevigata OR Crataegus oxyacantha OR mayflower^b^ | 31/01/2007 | 3 |
|  | hawthorn OR Crataegus laevigata OR Crataegus oxyacantha OR mayflower^c^ | 31/01/2007 | 13 |
| Hops | KW=(hops OR Humulus lupulus OR humulene OR lupulene) ^a^ | 31/01/2007 | 10 |
|  | hops OR Humulus lupulus OR humulene OR lupulene^b^ | 31/01/2007 | 5 |
|  | hops OR Humulus lupulus OR humulene OR lupulene^c^ | 31/01/2007 | 28 |
| Hyssop | hyssop OR Hyssopus officinalis^a^ | 31/01/2007 | 2 |
|  | hyssop OR Hyssopus officinalis^b^ | 31/01/2007 | 19 |
|  | hyssop OR Hyssopus officinalis^c^ | 31/01/2007 | 0 |
| Lemongrass leaves | lemongrass OR lemon grass OR Cymbopogon citratus^a^ | 31/01/2007 | 0 |
|  | lemongrass OR lemon grass OR Cymbopogon citratus^b^ | 31/01/2007 | 0 |
|  | lemongrass OR lemon grass OR Cymbopogon citratus^c^ | 31/01/2007 | 4 |
| Licorice | licorice OR liquorice OR Glycyrrhiza glabra^a^ | 31/01/2007 | 17 |
|  | licorice OR liquorice OR Glycyrrhiza glabra^b^ | 31/01/2007 | 0 |
|  | licorice OR liquorice OR Glycyrrhiza glabra^c^ | 31/01/2007 | 11 |
| Milk thistle | milk thistle OR Silybum marianum OR Variegated Thistle^a^ | 31/01/2007 | 1 |
|  | milk thistle OR Silybum marianum OR Variegated Thistle^b^ | 31/01/2007 | 0 |
|  | milk thistle OR Silybum marianum OR Variegated Thistle^c^ | 31/01/2007 | 9 |
| Mistletoe | mistletoe OR Viscum album^a^ | 31/01/2007 | 6 |
|  | mistletoe OR Viscum album^b^ | 31/01/2007 | 7 |
|  | mistletoe OR Viscum album^c^ | 31/01/2007 | 7 |
| Motherwort | motherwort OR Leonurus cardiaca OR Throw-wort OR Lion's Ear OR Lion's Tail^a^ | 31/01/2007 | 1 |
|  | motherwort OR Leonurus cardiaca OR Throw-wort OR Lion's Ear OR Lion's Tail^b^ | 31/01/2007 | 0 |
|  | motherwort OR Leonurus cardiaca OR Throw-wort OR Lion's Ear OR Lion's Tail^c^ | 31/01/2007 | 2 |
| Nettles | KW=(nettle OR nettles OR Urtica dioica) ^a^ | 31/01/2007 | 7 |
|  | nettle OR nettles OR Urtica dioica^b^ | 31/01/2007 | 2 |
|  | nettle OR nettles OR Urtica dioica^c^ | 31/01/2007 | 3 |
| Oats | oats OR Avena sativa^a^ | 31/01/2007 | 27 |
|  | oats OR Avena sativa^b^ | 31/01/2007 | 2 |
|  | oats OR Avena sativa^c^ | 31/01/2007 | 23 |
| Peppermint | peppermint OR Mentha piperita^a^ | 31/01/2007 | 20 |
|  | peppermint OR Mentha piperita^b^ | 31/01/2007 | 6 |
|  | peppermint OR Mentha piperita^c^ | 31/01/2007 | 7 |
| Rehmannia | rehmannia OR shengdihuang OR Chinese Foxglove^a^ | 31/01/2007 | 0 |
|  | rehmannia OR shengdihuang OR Chinese Foxglove^b^ | 31/01/2007 | 0 |
|  | rehmannia OR shengdihuang OR Chinese Foxglove^c^ | 31/01/2007 | 7 |
| Schizandra | schi*andra OR wuweizi^a^ | 31/01/2007 | 2 |
|  | schi*andra OR wuweizi^b^ | 31/01/2007 | 0 |
|  | schi*andra OR wuweizi^c^ | 31/01/2007 | 5 |
| Skullcap | skullcap OR Scutellaria lateriflora^a^ | 31/01/2007 | 1 |
|  | skullcap[TIAB] OR Scutellaria lateriflora^b^ | 31/01/2007 | 1 |
|  | skullcap OR Scutellaria lateriflora^c^ | 31/01/2007 | 7 |
| Spirulina | spirulina OR Arthrospira platensis^a^ | 31/01/2007 | 0 |
|  | spirulina OR Arthrospira platensis^b^ | 31/01/2007 | 0 |
|  | spirulina OR Arthrospira platensis^c^ | 31/01/2007 | 1 |
| St ignatius bean | St Ignatius bean OR Ignatia amara^a^ | 31/01/2007 | 0 |
|  | St Ignatius bean OR Ignatia amara^b^ | 31/01/2007 | 0 |
|  | St Ignatius bean OR Ignatia amara^c^ | 31/01/2007 | 0 |
| Wild yam | wild yam OR Dioscorea villosa^a^ | 31/01/2007 | 2 |
|  | wild yam OR Dioscorea villosa^b^ | 31/01/2007 | 0 |
|  | wild yam OR Dioscorea villosa^c^ | 31/01/2007 | 0 |
| Wood betony | wood betony OR Stachys officinalis OR Betonica officinalis^a^ | 31/01/2007 | 0 |
|  | wood betony OR Stachys officinalis OR Betonica officinalis^b^ | 31/01/2007 | 0 |
|  | wood betony OR Stachys officinalis OR Betonica officinalis^c^ | 31/01/2007 | 0 |
| Zizyphus spinosa | zizyphus OR suan zao ren^a^ | 31/01/2007 | 2 |
|  | zizyphus OR suan zao ren^b^ | 31/01/2007 | 1 |
|  | zizyphus OR suan zao ren^c^ | 31/01/2007 | 2 |
| Thyme | thyme OR Thymus vulgaris^a^ | 31/01/2007 | 3 |
|  | thyme OR Thymus vulgaris^b^ | 31/01/2007 | 0 |
|  | thyme OR Thymus vulgaris^c^ | 31/01/2007 | 4 |
| Purslane | purslane OR Portulaca oleracea OR Verdolaga OR Pigweed OR Little Hogweed OR Pusley^a^ | 31/01/2007 | 2 |
|  | purslane OR Portulaca oleracea OR Verdolaga OR Pigweed OR Little Hogweed OR Pusley^b^ | 31/01/2007 | 0 |
|  | purslane OR Portulaca oleracea OR Verdolaga OR Pigweed OR Little Hogweed OR Pusley^c^ | 31/01/2007 | 1 |
| Rosemary | KW=(rosemary OR Rosmarinus officinalis)^a^ | 31/01/2007 | 12 |
|  | rosemary OR Rosmarinus officinalis^b^ | 31/01/2007 | 7 |
|  | rosemary OR Rosmarinus officinalis^c^ | 31/01/2007 | 11 |
| Sage | KW=(sage OR Salvia officinalis) ^a^ | 31/01/2007 | 41 |
|  | sage OR Salvia officinalis^b^ | 31/01/2007 | 30 |
|  | sage OR Salvia officinalis^c^ | 31/01/2007 | 23 |
| Clove | cloves OR Eugenia caryophyllata OR Syzygium aromaticum^a^ | 31/01/2007 | 19 |
|  | cloves OR Eugenia caryophyllata OR Syzygium aromaticum^b^ | 31/01/2007 | 0 |
|  | cloves OR Eugenia caryophyllata OR Syzygium aromaticum^c^ | 31/01/2007 | 2 |
| Basil | KW=(basil OR Ocimum)^a^ | 31/01/2007 | 6 |
|  | basil OR Ocimum^b^ | 31/01/2007 | 2 |
|  | basil OR Ocimum^c^ | 31/01/2007 | 4 |
| Withania somnifera | ashwagandha OR Withania somnifera^a^ | 31/01/2007 | 4 |
|  | ashwagandha OR Withania somnifera^b^ | 31/01/2007 | 1 |
|  | ashwagandha OR Withania somnifera^c^ | 31/01/2007 | 0 |
| Euphytose | euphytose^a^ | 31/01/2007 | 0 |
|  | euphytose^b^ | 31/01/2007 | 1 |
|  | euphytose^c^ | 31/01/2007 | 0 |
| Tension tamer | tension tamer^a^ | 31/01/2007 | 0 |
|  | tension tamer^b^ | 31/01/2007 | 0 |
|  | tension tamer^c^ | 31/01/2007 | 0 |
| Mindsoothe | mindsoothe^a^ | 31/01/2007 | 0 |
|  | mindsoothe^b^ | 31/01/2007 | 0 |
|  | mindsoothe^c^ | 31/01/2007 | 0 |
| Sedariston | Sedariston^a^ | 31/01/2007 | 1 |
|  | Sedariston^b^ | 31/01/2007 | 0 |
|  | Sedariston^c^ | 31/01/2007 | 0 |
| Empowerplus | KW=(Empowerplus OR empower+)^a^ | 1/02/2007 | 109 |
|  | truehope^a^ | 1/02/2007 | 1 |
|  | Empowerplus OR empower+ OR Em powerplus OR em power+ OR E.m.powerplus OR e.m.power+ OR truehope^b^ | 1/02/2007 | 22 |
|  | Empowerplus OR empower+ OR truehope^c^ | 1/02/2007 | 34 |
| Worry free | Worry Free OR Maharishi Ayurvedic^a^ | 1/02/2007 | 2 |
|  | Worry Free OR Maharishi Ayurvedic^b^ | 1/02/2007 | 0 |
|  | Worry Free OR Maharishi Ayurvedic^c^ | 1/02/2007 | 62 |
| Tissue salts | tissue salts OR Schuessler^a^ | 1/02/2007 | 98 |
|  | tissue salts OR Schuessler^b^ | 1/02/2007 | 15 |
|  | tissue salts OR Schuessler^c^ | 1/02/2007 | 69 |
| Yeast | yeast^a^ | 1/02/2007 | 108 |
|  | yeast^b^ | 1/02/2007 | 128 |
|  | yeast^c^ | 1/02/2007 | 45 |
| Zinc | KW=zinc^a^ | 1/02/2007 | 49 |
|  | zinc[TIAB]^b^ | 1/02/2007 | 153 |
|  | zinc^c^ | 1/02/2007 | 99 |
| Taurine | taurine OR 2-aminoethanesulfonic acid^a^ | 2/02/2007 | 111 |
|  | taurine OR 2-aminoethanesulfonic acid^b^ | 2/02/2007 | 52 |
|  | taurine OR 2-aminoethanesulfonic acid^c^ | 2/02/2007 | 15 |
| Chamomile | chamomile OR Anthemis nobilis OR ground apple OR whig plant OR camomile OR Chamaemelum nobile OR Matricaria recutita OR Matricaria suaveolens OR Chamomilla OR Scented Mayweed^a^ | 2/02/2007 | 14 |
|  | chamomile OR Anthemis nobilis OR ground apple OR whig plant OR camomile OR Chamaemelum nobile OR Matricaria recutita OR Matricaria suaveolens OR Chamomilla OR Scented Mayweed^b^ | 2/02/2007 | 8 |
|  | Chamaemelum nobile OR Matricaria recutita OR Matricaria suaveolens OR Chamomilla OR Scented Mayweed^c^ | 2/02/2007 | 0 |
|  | chamomile OR Anthemis nobilis OR ground apple OR whig plant OR camomile^c^ | 2/02/2007 | 5 |
| Chromium | chromium^a^ | 2/02/2007 | 53 |
|  | chromium^b^ | 2/02/2007 | 53 |
|  | chromium^c^ | 2/02/2007 | 13 |
| Coenzyme q10 | coenzyme q10 OR CoQ OR ubiquinone OR ubiquinol^a^ | 2/02/2007 | 70 |
|  | coenzyme q10 OR CoQ OR ubiquinone OR ubiquinol^b^ | 2/02/2007 | 20 |
|  | coenzyme q10 OR CoQ OR ubiquinone OR ubiquinol^c^ | 2/02/2007 | 7 |
| Passionflower | passionflower OR Passiflora incarnata^a^ | 2/02/2007 | 7 |
|  | passionflower OR Passiflora incarnata^b^ | 2/02/2007 | 2 |
|  | passionflower OR Passiflora incarnata^c^ | 2/02/2007 | 2 |
| Valerian | valerian OR Valeriana officinalis^a^ | 2/02/2007 | 90 |
|  | valerian OR Valeriana officinalis^b^ | 2/02/2007 | 26 |
|  | valerian OR Valeriana officinalis^c^ | 2/02/2007 | 5 |
| Berocca | berocca^a^ | 5/02/2007 | 2 |
|  | berocca^b^ | 5/02/2007 | 0 |
|  | berocca^c^ | 5/02/2007 | 1 |
| Lecithin | lecithin OR phosphatidylcholine^a^ | 5/02/2007 | 115 |
|  | lecithin OR phosphatidylcholine^b^ | 5/02/2007 | 75 |
|  | lecithin OR phosphatidylcholine^c^ | 5/02/2007 | 28 |
| Choline | KW=choline^a^ | 5/02/2007 | 160 |
|  | choline[TIAB]^b^ | 5/02/2007 | 170 |
|  | choline^c^ | 5/02/2007 | 28 |
| Potassium | KW=potassium^a^ | 5/02/2007 | 136 |
|  | potassium[TIAB]^‡b^ | 5/02/2007 | 119 |
|  | potassium^§c^ | 5/02/2007 | 24 |
| Kava | kava OR piper methysticum OR kawa OR kavain OR rauschpfeffer^a^ | 6/02/2007 | 146 |
|  | kava OR piper methysticum OR kawa OR kavain OR rauschpfeffer^b^ | 6/02/2007 | 24 |
|  | kava OR piper methysticum OR kawa OR kavain OR rauschpfeffer^c^ | 6/02/2007 | 11 |
| Bach flower remedies | bach flower OR flower remed* OR flower essences OR rescue remedy^a^ | 6/02/2007 | 8 |
|  | bach flower OR flower remed* OR flower essences OR rescue remedy^b^ | 6/02/2007 | 3 |
|  | bach flower OR flower remed* OR flower essences OR rescue remedy^c^ | 6/02/2007 | 12 |
| Magnesium | KW=magnesium^a^ | 6/02/2007 | 95 |
|  | magnesium[TIAB] ^‡b^ | 6/02/2007 | 87 |
|  | magnesium^§c^ | 6/02/2007 | 30 |
| PABA | PABA OR para-aminobenzoic acid OR 4-aminobenzoic acid OR vitamin Bx^a^ | 7/02/2007 | 7 |
|  | PABA OR para-aminobenzoic acid OR 4-aminobenzoic acid OR vitamin Bx^b^ | 7/02/2007 | 91 |
|  | PABA OR para-aminobenzoic acid OR 4-aminobenzoic acid OR vitamin Bx^c^ | 7/02/2007 | 3 |
| GABA | KW=(GABA OR Gamma-aminobutyric acid)^a^ | 7/02/2007 | 323 |
|  | GABA[TIAB] OR Gamma-aminobutyric acid[TIAB]^‡b^ | 7/02/2007 | 319 |
|  | GABA OR Gamma-aminobutyric acid^§c^ | 7/02/2007 | 7 |
| Chaste tree berry | chaste tree berry OR vitex agnus castus^a^ | 7/02/2007 | 8 |
|  | chaste tree berry OR vitex agnus castus^b^ | 7/02/2007 | 3 |
|  | chaste tree berry OR vitex agnus castus^c^ | 7/02/2007 | 4 |
| Melatonin | KW=melatonin^a^ | 7/02/2007 | 318 |
|  | melatonin[TIAB] ^‡b^ | 7/02/2007 | 241 |
|  | melatonin^c^ | 7/02/2007 | 15 |
| Painkillers | KW=(painkillers OR aspirin OR acetylsalicylic acid OR codeine OR methylmorphine OR paracetamol OR acetaminophen)^a^ | 7/02/2007 | 117 |
|  | painkillers[TIAB] OR aspirin[TIAB] OR "acetylsalicylic acid"[TIAB] OR codeine[TIAB] OR methylmorphine[TIAB] OR paracetamol[TIAB] OR acetaminophen[TIAB] ^‡b^ | 8/02/2007 | 198 |
|  | painkillers OR aspirin OR acetylsalicylic acid OR codeine OR methylmorphine^§c^ | 7/02/2007 | 69 |
|  | ibuprofen OR analgesic^§c^ | 15/05/2007 | 91 |
|  | KW=(ibuprofen OR analgesics)^a^ | 15/05/2007 | 164 |
|  | ibuprofen^b^ | 15/05/2007 | 54 |
| Phenylalanine | KW=phenylalanine^a^ | 8/02/2007 | 87 |
|  | phenylalanine[TIAB]^b^ | 8/02/2007 | 151 |
|  | phenylalanine^c^ | 8/02/2007 | 14 |
| Inositol | KW=inositol^a^ | 8/02/2007 | 138 |
|  | inositol[TIAB]^b^ | 8/02/2007 | 181 |
|  | inositol^c^ | 8/02/2007 | 18 |
| Black cohosh | black cohosh OR Actaea racemosa OR Cimicifuga racemosa OR Black bugbane OR Black snakeroot^a^ | 8/02/2007 | 19 |
|  | black cohosh OR Actaea racemosa OR Cimicifuga racemosa OR Black bugbane OR Black snakeroot^b^ | 8/02/2007 | 13 |
|  | black cohosh OR Actaea racemosa OR Cimicifuga racemosa OR Black bugbane OR Black snakeroot^c^ | 8/02/2007 | 6 |
| Ketogenic diet | ketogenic diet^a^ | 8/02/2007 | 40 |
|  | ketogenic diet^b^ | 8/02/2007 | 4 |
|  | ketogenic diet^c^ | 8/02/2007 | 5 |
| Avoid barley, rye, wheat | KW=(barley OR rye OR wheat OR gluten OR Hordeum vulgare OR Secale cereale OR Triticum)^a^ | 8/02/2007 | 18 |
|  | barley OR rye OR wheat OR gluten OR Hordeum vulgare OR Secale cereale OR Triticum^b^ | 8/02/2007 | 67 |
|  | barley OR rye OR Hordeum vulgare OR Secale cereale OR Triticum^c^ | 8/02/2007 | 23 |
|  | gluten OR wheat^c^ | 8/02/2007 | 27 |
| Dairy avoidance | dairy OR lactose^a^ | 9/02/2007 | 147 |
|  | (avoid* OR reduc* OR depriv*) AND KW=(milk OR cream OR cheese OR butter OR yogurt OR yoghurt)^a^ | 9/02/2007 | 64 |
|  | dairy OR lactose^b^ | 9/02/2007 | 69 |
|  | (avoid* OR reduce OR reduction OR depriv*) AND (milk OR cream OR cheese OR butter OR yogurt OR yoghurt)^b^ | 9/02/2007 | 46 |
|  | dairy^c^ | 9/02/2007 | 52 |
| Carbohydrate-rich protein-poor diet | KW=(carbohydrate AND diet)^a^ | 9/02/2007 | 66 |
|  | carbohydrate[TIAB] AND diet^b^ | 9/02/2007 | 120 |
|  | carbohydrate AND diet^§c^ | 9/02/2007 | 6 |
| Hydrotherapy | hydrotherapy OR hydropathy^a^ | 9/02/2007 | 16 |
|  | hydrotherapy OR hydropathy^b^ | 9/02/2007 | 6 |
|  | hydrotherapy OR hydropathy^c^ | 9/02/2007 | 32 |
|  | KW=(baths OR spa OR whirlpool OR sauna OR bath therapy)^a^ | 25/09/2007 | 46 |
|  | baths OR spa OR whirlpool OR sauna OR bath therapy^b^ | 25/09/2007 | 61 |
|  | Balneotherapy OR bath therapy^§c^ | 3/04/2007 | 7 |
|  | Balneotherapy OR bath therapy^a^ | 3/04/2007 | 9 |
|  | Balneotherapy OR bath therapy^b^ | 3/04/2007 | 40 |
| Sleep deprivation | DE=sleep deprivation^a^ | 12/02/2007 | 270 |
|  | sleep deprivation^c^ | 12/02/2007 | 25 |
|  | sleep deprivation^†b^ | 12/02/2007 | 156 |
|  | wake therapy^§c^ | 13/09/2007 | 5 |
|  | wake therapy^a^ | 13/09/2007 | 12 |
|  | wake therapy^b^ | 13/09/2007 | 2 |
|  | sleep loss^†a^ | 2/10/2007 | 76 |
|  | sleep loss^b^ | 2/10/2007 | 115 |
| 5-Hydroxytryptophan | 5-Hydroxytryptophan OR 5-HTP^c^ | 12/02/2007 | 4 |
|  | KW=(5-Hydroxytryptophan OR 5-HTP)^a^ | 12/02/2007 | 92 |
|  | 5-Hydroxytryptophan OR 5-HTP^b^ | 12/02/2007 | 193 |
| Smoking | DE=nicotine OR DE=nicotine withdrawal OR DE=tobacco smoking OR DE=smoking cessation^a^ | 22/03/2007 | 412 |
|  | "Smoking/psychology"[MeSH:NoExp] OR "Smoking/therapeutic use"[MeSH:NoExp] OR "nicotine/therapeutic use"[MeSH] OR "smoking cessation"[MeSH] ^‡b^ | 22/03/2007 | 329 |
|  | nicotine OR smoking OR tobacco^§c^ | 22/03/2007 | 88 |
| Bibliotherapy | bibliotherapy^c^ | 15/02/2007 | 20 |
|  | KW=(bibliotherapy OR self-help manual OR self-help book)^a^ | 15/02/2007 | 155 |
|  | bibliotherapy OR self-help manual OR self-help book^b^ | 15/02/2007 | 48 |
| Caffeine | KW=caffeine^a^ | 21/02/2007 | 319 |
|  | caffeine^§c^ | 21/02/2007 | 12 |
|  | caffeine^‡b^ | 21/02/2007 | 219 |
| Exercise | exercise^§c^ | 22/02/2007 | 90 |
|  | KW=(exercise OR physical activit*)^†a^ | 22/02/2007 | 100 |
|  | exercise[TI] OR physical activity[TI] ^†b^ | 22/02/2007 | 165 |
| Meditation | KW=meditat*^a^ | 23/02/2007 | 245 |
|  | meditate*^c^ | 23/02/2007 | 40 |
|  | meditat*^b^ | 23/02/2007 | 140 |
|  | mindfulness^c^ | 22/08/2007 | 8 |
|  | KW=mindfulness^a^ | 22/08/2007 | 364 |
|  | mindfulness[TIAB]^b^ | 22/08/2007 | 78 |
| Humour | humor OR humour OR comedy OR laugh*^c^ | 9/03/2007 | 34 |
|  | DE=(humor OR humour OR comedy OR laugh*)^a^ | 9/03/2007 | 269 |
|  | humor[TIAB] OR humour[TIAB] OR comedy[TIAB] OR laugh*[TIAB]^b^ | 9/03/2007 | 251 |
| Marijuana | marijuana OR cannabis OR marihuana^c^ | 15/03/2007 | 47 |
|  | DE=(marijuana OR cannabis OR marihuana)^a^ | 15/03/2007 | 278 |
|  | Marijuana[TIAB] OR cannabis[TIAB] OR marihuana[TIAB]^‡b^ | 15/03/2007 | 170 |
| Pleasant activities | pleasant activit*^c^ | 20/03/2007 | 17 |
|  | pleasant activit*^b^ | 20/03/2007 | 24 |
|  | pleasant activit*^a^ | 21/03/2007 | 283 |
|  | "pleasant events" OR "activity scheduling" OR "behavio?ral activation"^b^ | 2/08/2007 | 118 |
|  | KW=("pleasant events" OR "activity scheduling" OR "behavio?ral activation")^a^ | 2/08/2007 | 154 |
|  | "pleasant events" OR "activity scheduling" OR "behavio?ral activation"^*c^ | 2/08/2007 | 7 |
| Recreation | "Recreation/psychology"[MeSH] OR "Recreation/therapy"[MeSH] ^b^ | 20/03/2007 | 262 |
|  | DE=(hobbies OR recreation OR leisure time)^a^ | 21/03/2007 | 285 |
|  | hobbies OR recreation OR leisure^*c^ | 21/03/2007 | 4 |
| Distraction | distraction^c^ | 21/03/2007 | 42 |
|  | DE=distraction^a^ | 21/03/2007 | 125 |
|  | distraction^b^ | 21/03/2007 | 200 |
| Relaxation | relaxation^*c^ | 26/03/2007 | 44 |
|  | DE=relaxation^a^ | 26/03/2007 | 451 |
|  | relaxation[MESH] OR "relaxation techniques"[MESH]^b^ | 26/03/2007 | 287 |
| Reflexology | reflexology OR zone therapy^*c^ | 27/03/2007 | 4 |
|  | reflexology OR zone therapy^a^ | 27/03/2007 | 33 |
|  | reflexology OR zone therapy^b^ | 27/03/2007 | 115 |
| Autogenic training | autogenic training OR autogenics^c^ | 27/03/2007 | 26 |
|  | DE=(autogenic training OR autogenics)^a^ | 27/03/2007 | 24 |
|  | autogenic training OR autogenic^b^ | 27/03/2007 | 38 |
| Massage | massage^*c^ | 28/03/2007 | 33 |
|  | KW=massage^a^ | 28/03/2007 | 123 |
|  | massage^b^ | 28/03/2007 | 171 |
| Saffron | saffron OR Crocus sativus^c^ | 3/04/2007 | 1 |
|  | saffron OR Crocus sativus^a^ | 3/04/2007 | 31 |
|  | saffron OR Crocus sativus^b^ | 3/04/2007 | 6 |
| Crystal healing | crystal healing OR charmstone^c^ | 3/04/2007 | 7 |
|  | crystal healing OR charmstone^a^ | 3/04/2007 | 2 |
|  | crystal healing OR charmstone^b^ | 3/04/2007 | 0 |
| Pilates | pilates^c^ | 3/04/2007 | 1 |
|  | pilates^a^ | 3/04/2007 | 0 |
|  | pilates^b^ | 3/04/2007 | 0 |
| Qigong | qigong^c^ | 3/04/2007 | 6 |
|  | qigong^a^ | 3/04/2007 | 65 |
|  | qigong^b^ | 3/04/2007 | 58 |
| Computerised interventions | KW=((computer* OR internet) AND (CBT OR bibliotherapy))^a^ | 26/07/2007 | 44 |
|  | internet OR computer OR CBT AND depressi*^c^ | 26/07/2007 | 129 |
|  | (computer* OR internet) AND (CBT OR bibliotherapy)^b^ | 26/07/2007 | 28 |
| Rhodiola rosea | Rhodiola rosea OR roseroot OR golden root^c^ | 30/07/2007 | 3 |
|  | Rhodiola rosea OR roseroot OR golden root^a^ | 30/07/2007 | 2 |
|  | Rhodiola rosea OR roseroot OR golden root^b^ | 30/07/2007 | 1 |
| Lavender | lavender OR Lavandula^c^ | 30/07/2007 | 15 |
|  | KW=(lavender OR Lavandula) ^a^ | 30/07/2007 | 38 |
|  | lavender OR Lavandula^b^ | 30/07/2007 | 25 |
| Carnitine | carnitine^c^ | 30/07/2007 | 20 |
|  | carnitine^a^ | 30/07/2007 | 96 |
|  | carnitine^b^ | 30/07/2007 | 59 |
| Holiday | holiday OR vacation OR resort OR time off OR break^*c^ | 27/09/2007 | 110 |
|  | KW=(holiday OR vacation OR time off)^a^ | 27/09/2007 | 137 |
|  | Holiday[TIAB] OR vacation[TIAB] OR “time off”[TIAB]^b^ | 27/09/2007 | 79 |
| Shopping | shopping OR consumerism OR retail therapy^c^ | 27/09/2007 | 74 |
|  | KW=(shopping OR consumerism OR retail therapy)^a^ | 27/09/2007 | 271 |
|  | shopping OR consumerism OR retail^b^ | 27/09/2007 | 78 |
| Singing | singing OR choir^c^ | 27/09/2007 | 47 |
|  | singing OR choir^b^ | 27/09/2007 | 28 |
|  | KW=(singing OR choir)^a^ | 27/09/2007 | 86 |

1. ^§^a. PsycINFO

   b. PubMed

   c. Cochrane

   ^§^ Limited to title, abstract, keyword [↑](#footnote-ref-2)
2. ^†^ Limited to review or meta-analysis

   ^‡^ Limited to clinical trial, letter, meta-analysis, randomised controlled trial, review [↑](#footnote-ref-3)
3. ^†^ [↑](#footnote-ref-4)
